# Process optimizations for the synthesis of an intermediate of dydrogesterone†

**DOI:** 10.1039/d5ra00109a

**Published:** 2025-03-10

**Authors:** Zhongyue Wang, Yuan Wang, Shaoxiong Jing, Xiangjian Huang, Hucheng Wu, Yingquan Yang, Jian Song, Bao Zhang

**Affiliations:** a Tianjin University, School of Chemical Engineering and Technology Tianjin China yue7946801@tju.edu.cn baozhang@tju.edu.cn songjian@tju.edu.cn; b Suzhou Entai New Materials Technology Company Suzhou China huangxj0703@sina.com 375831573@qq.com

## Abstract

Dydrogesterone (DG) is a potent progesterone drug that can be used to treat almost all progesterone-deficiency in women. However, there are few reports on DG process studies, and the by-products are unclear, which make the process optimization challenging. In this paper, we report the process optimization studies for the synthesis of 9α,10β-pregest-5,7-diene-3,20-diethylene glycol ketone (1), an intermediate of DG. Starting from the natural raw progesterone, the intermediate 1 was synthesised *via* a three-step process involving ketal protection, allylic bromination and elimination. We synthesised and characterised the main by-products in the process route, and explored the effects of conditions such as feed amount, temperature and types of bases on the yield and selectivity. Compared with traditional thermal initiation, we employed a 365 nm LED lamp to initiate the allylic bromination, avoiding the need to add initiators during thermal initiation. The photoinduced reaction time was markedly diminished from 1.5 h to 20 min, while maintaining a bromination yield of over 65%. The optimized process route of intermediate 1 is featured with simple operation, short time, low energy consumption, few by-products, and easy to scale up production, which is critical for enhancing the production efficiency and reducing the environmental impact of DG.

## Abstract 2

This works develops an efficient three-step synthesis of dydrogesterone intermidiate 9α,10β-pregest-5,7-diene-3,20-diethylene glycol ketone through ketal production, innovative photoinitiated allylic bromination, and elimination.

## Graphical abstract

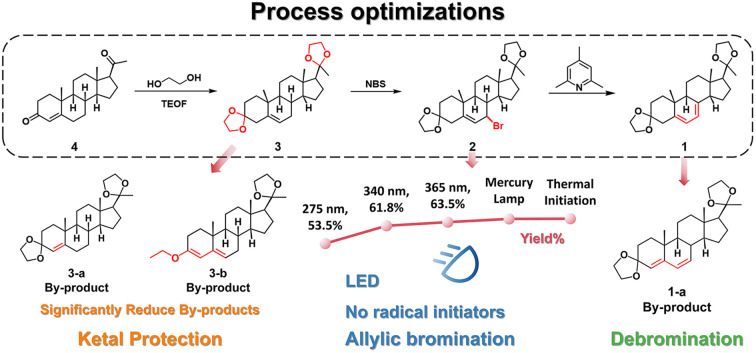

## 1 Introduction

Progesterone is a type of progestogen secreted by the corpus luteum cells of the ovary. Based on molecular structure, progestogens can be primarily divided into two main categories: one comprises testosterone and its structural analogs, and the other comprises progesterone and its structurally similar derivatives.^1,2^ Progesterone is a hormone of particular importance to women, but natural progesterone has low bioavailability after liver metabolism and has side effects such as dizziness.^3–6^ Thus various synthetic progestogen drugs have been developed.

**Fig. 1:**
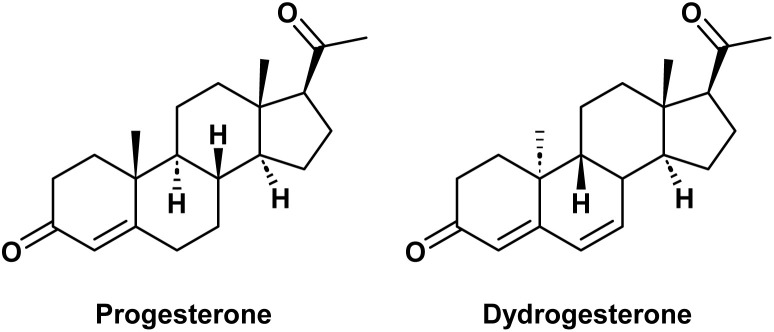
Molecular structures of progesterone and dydrogesterone.

Dydrogesterone (9β,10α-pregna-4,6-diene-3,20-dione, DG) is a potent progestogen with a structure similar to progesterone (Fig. 1). This drug was developed and marketed in 1961 by the SOLVAY, with the original drug name Duphaston.^7^ DG is an effective treatment for diseases associated with progesterone deficiency in women. It has high bioavailability, allowing for oral administration,^8,9^ and exhibits robust progestogenic activity while displaying minimal interaction with other hormone receptors in the body,^10,11^ such as estrogen,^12,13^ androgen,^2^ glucocorticoid (GC)^14^ and mineralocorticoid (MC). Consequently, DG is a progestogen drug with good therapeutic effect, promising development potential, and minimal adverse effects in clinical practice.^15–19^

Dydrogesterone can be synthesized using four different steroid substrates (Fig. S1–S5, ESI†). (1) The first method uses 3β-hydroxy-9α,10β-pregna-5-en-20-one (pregnenolone) as the starting material, going through oxidation, ketal protection, bromination, elimination, photoisomerization, and rearrangement to obtain the product DG, with an estimated yield of up to 60%. However, the reaction route is lengthy, and the process is challenging to control, thus it has not been widely applied.^20,21^ (2) The second method uses *trans*-progesterone as the starting material, synthesizing DG under nitrogen protection with tetrachlorobenzoquinone as the oxidant. The synthesis route is relatively brief, but *trans*-progesterone is not a natural product.^22^ (3) The third method employs ergosterol as the starting material, undergoing a six-step synthesis involving photoisomerization, biofermentation, rearrangement, oxidation, enamine formation, and oxidation. The biofermentation technology has mild reaction conditions, however the molar yield is relatively low, and industrial scale-up is difficult.^23^ (4) The fourth method, based on progesterone, is the main synthetic route for current production. It has two principal synthesis routes. One involves ketal protection, oxidation, hydrazone formation, dehydrazonation, photoisomerization, and rearrangement to prepare DG. However, the dehydrazonation process typically involves the use of lithium hydride, which is dangerous in large-scale production and prone to explosion;^24^ the other is through ketal protection, allylic bromination, debromination, photoisomerization, and hydrolytic rearrangement to prepare DG.^25^ This synthesis route has easily available raw materials, fewer reaction steps and easy to scale up. Therefore, we choose this route to prepare DG.

**Scheme 1:**
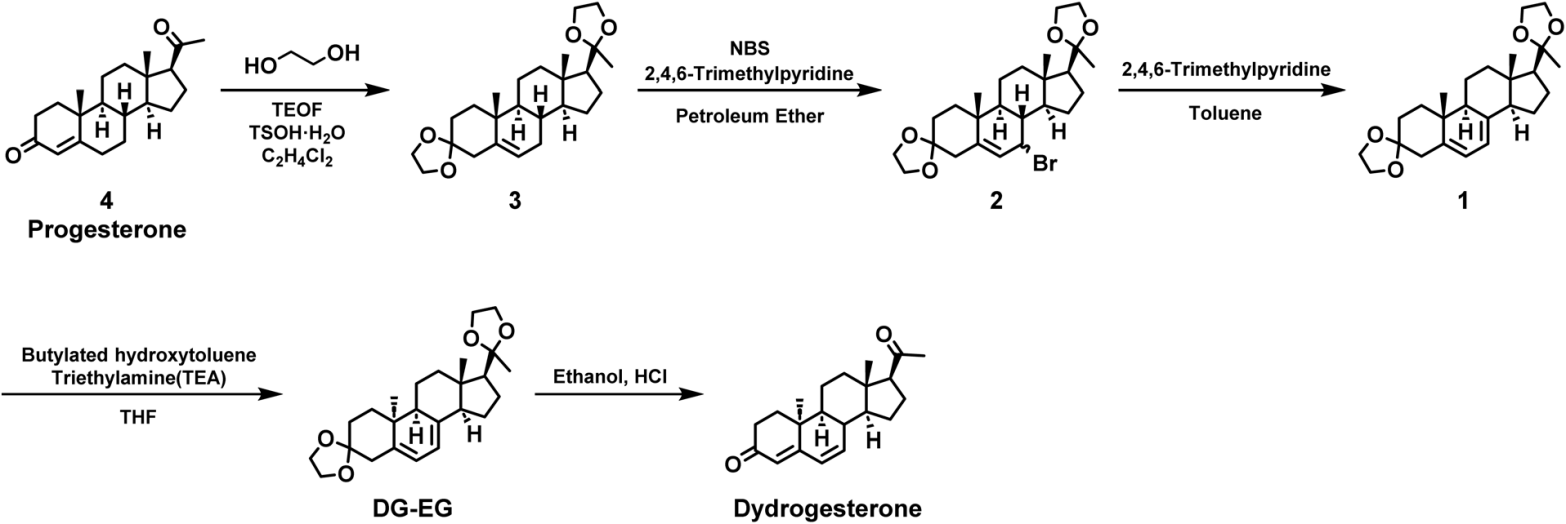
The synthetic route of DG intermediate 1.

9α,10β-Pregest-5,7-diene-3,20-diethylene glycol ketone (DG intermediate 1) is the key intermediate of this route (Scheme 1). The synthesis process frequently produces multiple by-products with ambiguous structures, making process optimization difficult. In this paper, we synthesized and structurally identified the principal impurities or reaction intermediates in the process route, and we systematically investigated the yield and proportion of by-products under various reaction conditions in order to optimize the process parameters for the three-step reaction. Furthermore, for the second step of the free radical reaction, we adopted photoinduced initiation, a method distinct from thermal initiation, enabling the reaction to be completed within 20 minutes. The optimized process is more energy-efficient and generates fewer by-products.

## 2 Results and discussion

### 2.1 Screening of reaction conditions for compound 3

**Fig. 2:**
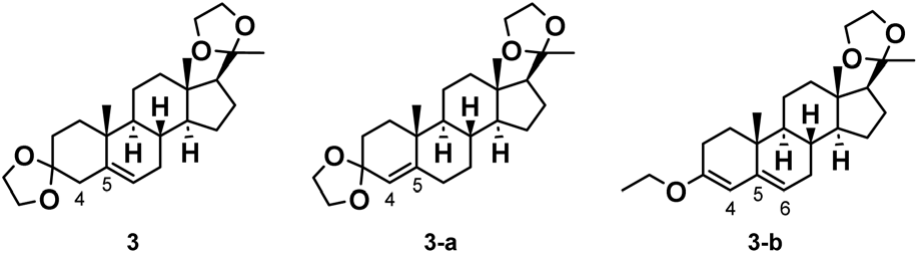
Structural comparison of the target product 3, Isomer 3-a and by-product 3-b.

The first step of the synthetic route entails the protection of the 3- and 20-carbonyl groups with ethylene glycol (EG), while the double bond migrates from the 4-position to the 5-position, leading to the desired product 3. The migration of the double bond is primarily influenced by the p*K*_a_ of the acid catalyst,^26,27^ therefore a certain proportion of the isomer product 3-a with the double bond not migrated is present in the reaction system (Fig. 2).

During the ketal protection reaction, each equivalent of progesterone produces two equivalents of water. The primary function of triethyl orthoformate (TEOF) is to facilitate dehydration, thereby promoting the reversible reaction to proceed in the desired direction. However, TEOF can react with the carbonyl group in compound 4 to form an enol ether, resulting in a certain proportion of by-products 3-b (Fig. 2). The structure of this by-product was determined by NMR and MS.

Therefore, in order to enhance the reaction yield and minimize the quantity of by-products, it is essential to optimize the reaction conditions for this step, including the quantity of EG fed, reaction temperature and time.

The feed equivalent of EG was first evaluated, with the results presented in Table 1. As can be observed, when the mole ratio of *n*(EG) : *n*(4) was less than 3 : 1, the ketal protection reaction was insufficient, resulting in the formation of the main by-product 3-b (Table 1, entries 1–2). The carbonyl group at the 20-position, due to the less steric hindrance, preferentially underwent ethylene glycol ketal protection reaction. However, the quantity of EG was not significantly excessive compared to TEOF. Thus the two reagents competed for the reaction site of the ketone carbonyl group at position 3, resulting in the formation of the by-product 3-b. Increasing the ratio of *n*(EG) : *n*(4) rapidly increased the yield of 3 (Table 1, entries 2–5). But after the mole ratio of *n*(EG) : *n*(4) exceeded 6 : 1, the yield began to decline, and accompanied by an increase in the number of impurities in the reaction system (Table 1, entries 6–9). Therefore, the optimal mole ratio was determined to be *n*(EG) : *n*(4) = 6 : 1 (Table 1, entry 5).

**Table 1:** Screening of ethylene glycol feeding equivalent

Entry^a^	EG : TEOF : 4 (equiv.)	Temp.	Time	Yield of 3^b^
1	2.0 : 2.3 : 1	40 °C	15 h	35.1%
2	3.0 : 2.3 : 1	40 °C	15 h	69.4%
3	4.0 : 2.3 : 1	40 °C	15 h	71.4%
4	5.0 : 2.3 : 1	40 °C	15 h	76.7%
5	6.0 : 2.3 : 1	40 °C	15 h	83.8%
6	7.0 : 2.3 : 1	40 °C	15 h	82.9%
7	8.0 : 2.3 : 1	40 °C	15 h	81.3%
8	9.0 : 2.3 : 1	40 °C	15 h	78.6%
9	10.0 : 2.3 : 1	40 °C	15 h	75.9%

a Conditions: compound 4 (63.60 mmol, 1 equiv.), TEOF (148.25 mmol, 2.3 equiv.), TsOH·H_2_O (2.10 mmol, 0.03 equiv.).

b Isolated yield after workup.

The ketal protection process is a reversible reaction, and the reaction temperature or time could affect the selectivity and conversion rate. Thus, the yield changes of compound 3 under various conditions were shown in Table 2. It can be observed that as the reaction temperature increased (Table 2, entries 1–5) and the time was extended (Table 2, entries 6–9, 3 and 10–12), the yield of 3 first increased and then decreased. The highest yield of 88.8% was achieved at a reaction temperature of 40 °C and a reaction time of 15 h (Table 2, entry 3). We further monitored the changes in the content of impurities and main products during the reaction process with different temperatures using HPLC (Table 3). When the temperature was too low, the reaction progress was insufficient, resulting in the production of significant quantities of impurity 3-b (Table 3, entries 1–2). When the temperature was too high, the amount of the by-product 3-a increased (Table 3, entries 4–5). The influence of time on the reaction process was analogous to that of temperature (Table S5, ESI†). Therefore, the optimal reaction conditions for the ketal protection process were determined to be 40 °C and 15 h.

**Table 2:** Screening of temperature and time

Entry^a^	Temp.	Time	Yield of 3^b^
1	20 °C	15 h	66.4%
2	30 °C	15 h	80.6%
3	40 °C	15 h	88.8%
4	50 °C	15 h	69.1%
5	60 °C	15 h	64.1%
6	40 °C	3 h	36.6%
7	40 °C	6 h	51.6%
8	40 °C	9 h	72.5%
9	40 °C	12 h	80.1%
10	40 °C	18 h	78.6%
11	40 °C	21 h	75.2%
12	40 °C	24 h	73.2%

a Conditions: compound 4 (63.60 mmol, 1 equiv.), EG (381.02 mmol, 6 equiv.), TEOF (148.25 mmol, 2.3 equiv.), TsOH·H_2_O (2.10 mmol, 0.03 equiv.).

b Isolated yield after workup.

**Table 3:** Variation of the content of product 3 and by-products 3-a and 3-b with different reaction temperature

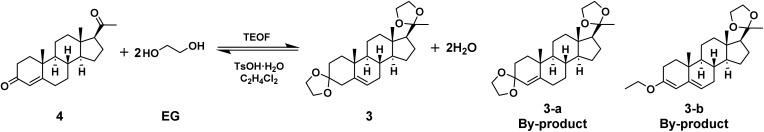				
Entry^a^	Temp.	3, wt%	3-a, wt%	3-b, wt%
1	20 °C	43.72	6.98	47.36
2	30 °C	65.32	6.32	21.10
3	40 °C	80.58	6.08	4.46
4	50 °C	81.97	7.28	4.78
5	60 °C	78.53	9.22	8.53

a Conditions: compound 4 (63.60 mmol, 1 equiv.), EG (381.02 mmol, 6 equiv.), TEOF (148.25 mmol, 2.3 equiv.), TsOH·H_2_O (2.10 mmol, 0.03 equiv.).

### 2.2 Screening of reaction conditions for compound 2

In free radical reactions, low concentrations of bromine can be provided by *N*-bromosuccinimide (NBS) continuously and stably.^28,29^ The free radical reaction could be initiated by light or heat, and different initiation methods can affect the rate, selectivity, and yield of this reaction.^30–34^ Therefore, we compared the influence of different initiation methods on the yield of compound 2.

First, we investigated whether light can initiate the allylic bromination of compound 3 and the initiation temperature (Table 4). Under the same reaction time, LED light or high-pressure mercury lamp was used for light induced reaction, and the yield was compared at the reaction temperature of room temperature or 60 °C. It was clear that the free radical reaction could be triggered by light (Table 4, entries 2, 5 and 6), and adding 3–5% initiator could increase the yield (Table 4, entries 3, 4, 7 and 8). However, the activation energy required for the reaction between bromine radicals and the raw material compound 3 was high, so that the temperature of the reaction solution must reach 60 °C or above to have the reaction to occur or increase the product yield. Thus, low temperature was not conducive to the allylic bromination reaction (Table 4, entries 1, 3, 5 and 7).

**Table 4:** Screening of initiation temperature and light source

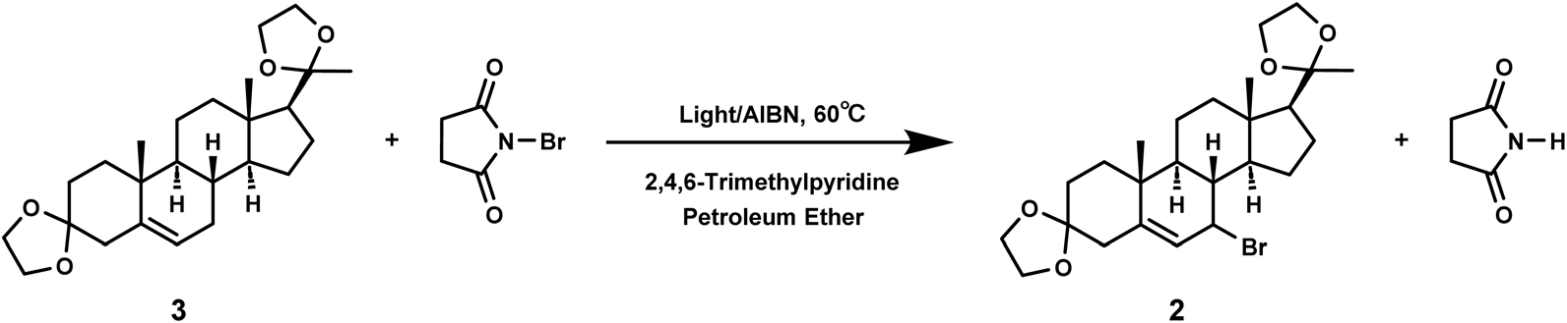					
Entry^a^	Light source	Initiator	Temp.	Time	Yield of 2^b^
1	LED, 340 nm	—	25 °C	90 min	0
2	LED, 340 nm	—	60 °C	90 min	61.8%
3	LED, 340 nm	AIBN (0.03 equiv.)	25 °C	90 min	1.3%
4	LED, 340 nm	AIBN (0.03 equiv.)	60 °C	90 min	62.2%
5	High pressure mercury lamp	—	25 °C	90 min	33.6%
6	High pressure mercury lamp	—	60 °C	90 min	57.0%
7	High pressure mercury lamp	AIBN (0.03 equiv.)	25 °C	90 min	46.6%
8	High pressure mercury lamp	AIBN (0.03 equiv.)	60 °C	90 min	60.6%

a Conditions: compound 3 (49.68 mmol, 1 equiv.), NBS(62.93 mmol, 1.3 equiv.).

b Isolated yield after workup.

Further investigation on the influence of wavelength on light induced reactions was performed. LED light sources can emit light of a single wavelength and release much less heat than high-pressure mercury lamps, and thus LED lamps were used to compare the effects of different wavelengths on the reaction efficiency (Table 5, entries 1–3). We selected LED light sources with wavelengths of 275, 340, and 365 nm, without the addition of initiators, and the reaction was performed at 60 °C for 90 minutes. The choice of wavelength may be related to the UV absorption of the substrate. The maximum absorption wavelengths of compound 3 and NBS are known to be at 331 nm and 349 nm respectively. Therefore, compared with thermal initiation (Table 5, entry 8), both 340 nm and 365 nm could promote the reaction, with a yield of over 60% achieved (Table 5, entries 2–3).

**Table 5:** Screening of wavelength of LED light and reaction time

Entry^a^	Light source	Wavelength	Initiator	Temp.	Time	Yield of 2^b^
1	LED	275 nm	—	60 °C	90 min	53.5%
2	LED	340 nm	—	60 °C	90 min	61.8%
3	LED	365 nm	—	60 °C	90 min	62.9%
4	LED	340 nm	—	60 °C	20 min	6.5%
5	LED	365 nm	—	60 °C	20 min	63.5%
6	High pressure mercury lamp	Characteristic spectrum	—	60 °C	20 min	65.0%
7	High pressure mercury lamp	Characteristic spectrum	—	60 °C	90 min	57.0%
8	—	—	—	60 °C	90 min	58.2%
9	—	—	AIBN (0.03 equiv.)	60 °C	90 min	65.1%
10	—	—	—	60 °C	20 min	1.1%
11	—	—	AIBN (0.03 equiv.)	60 °C	20 min	43.9%

a Conditions: compound 3 (49.68 mmol, 1 equiv.), NBS(62.93 mmol, 1.3 equiv.).

b Isolated yield after workup.

During light-induced reactions, the solution color changed significantly after 20 minutes of light exposure, indicating a faster reaction rate than thermal reaction. Therefore, we attempted to evaluate the reaction time under light initiation, and compared the results at 60 °C for 20 and 90 minutes (Table 5, entries 2–11). It was challenging to complete the allylic bromination in 20 minutes by thermal initiation (Table 5, entries 10 and 11), whereas the bromination could be essentially completed within 20 minutes under photoinitiation (Table 5, entries 5 and 6). It was shown that upon heating at 60 °C, with the LED initiation at the 365 nm wavelength and no initiator added, the reaction basically occurred within 15–20 minutes, with a yield of 63.5%. Continuing to extend the reaction time may lead to increased side reactions, resulting in a decreased yield of compound 2 (Table 5, entries 3, 5, 6 and 7).

It could be concluded that the yield of bromination was greater when induced by rapid photoinitiation with 365 nm LED light or high-pressure mercury lamp, or traditional thermal initiation with AIBN (Table 5, entries 5, 6 and 9). However, high pressure mercury lamps have high power and large heat release, which may pose certain safety hazards in actual production.^35^ The yield of LED photoinitiation at 365 nm was comparable to that of traditional thermal initiation, but the former was more time- and energy-efficient, with no requirement of the addition of free radical initiators, and was therefore more suitable for industrial-scale production.^36^

### 2.3 Screening of reaction conditions for compound 1

**Fig. 3:**
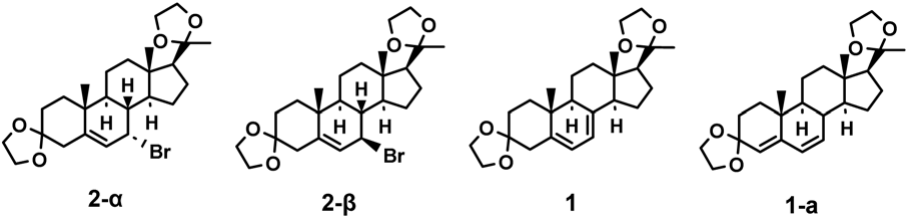
Structural comparison of compounds 2-α, 2-β, 1 and 1-a.

The product 2 contains chiral isomers 2-α and 2-β as shown in Fig. 3. The elimination process for the brominated compounds with different configurations is distinct, resulting in different conjugated diene products 1 and 1-a.^37–39^ The target product 1 can be obtained by E2 elimination of 2-α, and the main by-product is determined to be 3,20-di(ethylenedioxy)pregnen-4,6-diene 1-a. We thus further investigated how the different type of base and reaction time would influence the selectivity of the elimination reaction, and the results were summarized in Table 6.

**Table 6:** Screening of bases

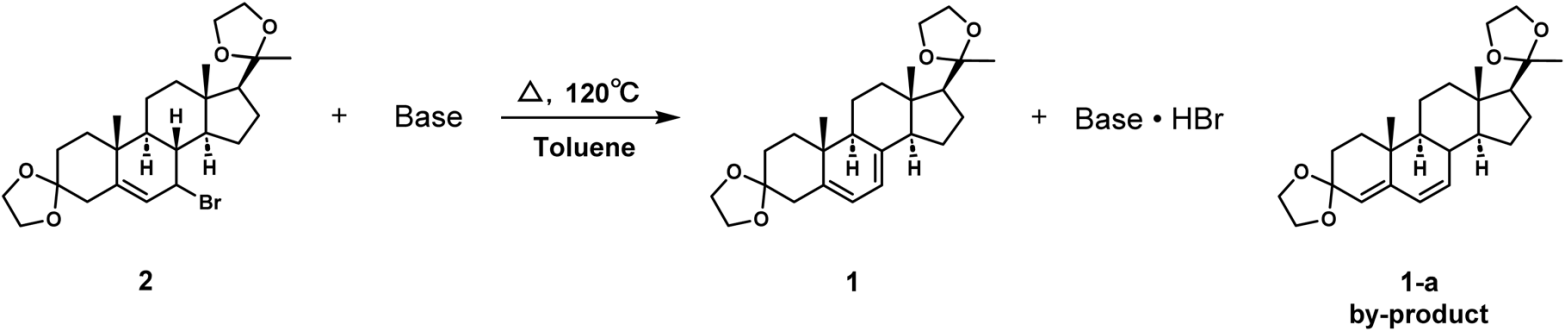			
Entry^a^	Base	1-a : 1 (HPLC area%)	Yield of 1^b^
1	DBU	14.88 : 1	4.0%
2	TBAF·3H_2_O	1.96 : 1	8.6%
3	TBAF·THF	0.61 : 1	26.3%
4	DIPEA	0.55 : 1	39.8%
5	TEA	0.38 : 1	55.7%
6	2,6-Dimethylpyridine	0.27 : 1	80.8%
7	2,4,6-Trimethylpyridine	0.25 : 1	84.2%

a Conditions: compound 2 (31.53 mmol, 1 equiv.), base (2.4 equiv.).

b Isolated yield after workup.

It was found that when NaOH, CH_3_ONa (Fig. S21 and S22, ESI†) and tetrabutylammonium fluoride (Table 6, entries 2–3) were employed, almost no products generated. It was speculated that when the nucleophilicity of the base was strong or the polarity of the solvent increased, substitution reactions could occur preferentially, producing alkyl substitution by-products. When using amines and DBU as organic bases, 4,6-diene impurities 1-a were mainly obtained (Table 6, entries 1, 4 and 5). This was hypothesized to be associated with the alkaline strength of the organic base. The pH of DBU, TEA, and DIPEA in toluene or aqueous solutions is greater than 11, while the pH of 2,6-dimethylpyridine and 2,4,6-trimethylpyridine is 7–9 (Table S6, ESI†). Therefore, the alkalinity of DBU, TEA, and DIPEA is significantly higher than that of 2,6-dimethylpyridine and 2,4,6-trimethylpyridine. Impurities 1-a will be mainly obtained when the alkalinity is too strong. Therefore, 2,4,6-trimethylpyridine proved to be the best choice for the reaction.

We then explored the effect of reaction time on the yield of 1. As illustrated in Table 7, extended reaction time resulted in the more complete conversion of compound 2, yielding a greater quantity of eliminated products (Table 7, entries 1 and 6). However, the yield of product 1 decreased when the reaction time exceeded 1.5 h (Table 7, entries 4–6). The elimination process of compound 2 was governed by two competing pathways. The first was the trans coplanar elimination, which involved the hydrogen at position 8 and the bromine at position 7, resulting in the formation of the desired product 1. The other occurred *via* the carbocation migration, followed by nucleophilic attack on the β-H at position 4, resulting in the by-product 4,6-diene impurity 1-a. Stability analysis was conducted on pure product 1 under reaction conditions, and it was found that the ratio of product 1 to 4,6-diene 1-a decreased by an average of 0.5% every 1 h (Table S7, ESI†). Consequently, if the reaction time had exceeded 1.5 h, there might have been a reversible conversion between product 1 and by-product 1-a. Thus, 1.5 h was determined to be the optimum elimination reaction time.

**Table 7:** Screening of reaction time

Entry^a^	Time	Eliminated products^b^ : 2 (HPLC area%)	Yield of 1^c^
1	0.5 h	5.6	63.1%
2	1.0 h	19.4	70.0%
3	1.5 h	28.8	75.9%
4	2.0 h	39.2	69.6%
5	3.0 h	43.4	70.2%
6	4.0 h	46.8	65.1%

a Conditions: compound 2 (31.53 mmol, 1 equiv.), 2,4,6-trimethylpyridine(2.4 equiv.).

b Eliminated products are compounds 1 and 1-a.

c Isolated yield after workup.

## 3 Conclusions

In this article, the key intermediate 1 of DG was prepared from cheap and readily available progesterone by ketal protection, allylic bromination, and debromination. The critical impurities in the reaction processes such as ketal protection have been synthesized and characterized. By analyzing the changes in the content of by-products, we optimized the reaction conditions of this synthetic route in detail. The key allylic bromination reaction was conducted using LED photoinitiation of specific wavelengths without the need of a free radical initiator and lengthy reaction times. The optimized route has greatly reduced the proportion of by-products and the energy consumption of the reaction process, making it more environmentally friendly. The target product was produced in a total molar yield of over 60%, and thus the route has the potential for large-scale production.

## 4 Experimental section

### 4.1 Chemistry general methods

All commercially available materials and solvents were used directly without further purification. The reactions were monitored by a Shimadzu LC-20AT HPLC instrument. The ^1^H NMR and ^13^C NMR spectra were recorded using a Bruker Advance 400 MHz nuclear magnetic resonance (NMR) spectrometer with tetramethylsilane (TMS) as an internal standard. The mass spectra were recorded on a Shimadzu LC-MS 2020.

### 4.2 9α,10β-Pregna-5-ene-3,20-diethylene glycol ketone (3)

To a solution of progesterone 4 (20.00 g, 63.60 mmol, 99.08% pure) in a mixture of solvent dichloroethane (60.00 g), triethyl orthoformate (22.00 g, 148.25 mmol) and ethylene glycol (23.65 g, 381.02 mmol) was added *p*-toluenesulfonic acid monohydrate (0.40 g, 2.10 mmol) at room temperature, the reaction mixture was allowed to heat to 40 °C and stir for 15 h. The reaction was cooled down to 0 °C. The crude product was filtered and the filter cake was recrystallized from ethyl acetate (60.00 g). The target product 3 (22.74 g, 88.8%) was obtained. ^1^H NMR (400 MHz; CDCl_3_) *δ* 5.34 (dt, *J* = 4.9, 2.2 Hz, 1H), 4.05–3.82 (m, 8H), 2.56 (dq, *J* = 14.2, 3.0 Hz, 1H), 2.11 (dd, *J* = 14.1, 2.9 Hz, 1H), 2.06 (dt, *J* = 12.6, 3.5 Hz, 1H), 1.96 (dtd, *J* = 17.6, 5.3, 2.9 Hz, 1H), 1.86–1.38 (m, 12H), 1.34 (dd, *J* = 13.6, 3.6 Hz, 1H), 1.29 (s, 3H), 1.25–1.10 (m, 2H), 1.10–0.96 (m, 5H), 0.77 (s, 3H). ^13^C NMR (101 MHz; CDCl_3_) *δ* 209.28, 199.42, 170.92, 123.94, 77.36, 77.05, 76.73, 63.51, 56.03, 53.66, 43.92, 38.67, 38.58, 35.73, 35.56, 33.95, 32.78, 31.90, 31.49, 24.36, 22.84, 21.02, 17.38, 13.33. ESI-MS, *m*/*z*[M + H]^+^ found: 403.30.

### 4.3 7-Bromo-3,20-bis(vinylenedioxy)pregnenolone (2)

To a solution of compound 3 (20.00 g, 49.68 mmol) in a mixture of solvent petroleum ether (200.00 g) and 2,4,6-trimethylpyridine (9.20 g, 75.92 mmol) was added NBS (11.20 g, 62.93 mmol) at room temperature, the reaction mixture was allowed to heat to 60 °C under nitrogen protection. The mixture was illuminated with 365 nm LED light and stirred for 20 min. The reaction solution was cooled down to room temperature and concentrated under reduced pressure until no liquid dripped out. The concentrate was washed twice with toluene and water at room temperature, and dried over Na_2_SO_4_. After vacuum concentration, the target product 2 (15.18 g, 63.5%) was obtained. ^1^H NMR (400 MHz; Toluene-*d*_8_) *δ* 5.58 (dd, *J* = 5.3, 2.0 Hz, 1H), 4.45 (ddd, *J* = 5.4, 3.5, 2.1 Hz, 1H), 3.58–3.44 (m, 8H), 2.53 (dt, *J* = 14.1, 2.2 Hz, 1H), 1.94–1.48 (m, 12H), 1.42–1.35 (m, 2H), 1.20 (s, 3H), 1.13 (tt, *J* = 12.2, 3.7 Hz, 2H), 1.08–1.00 (m, 1H), 0.83 (s, 3H), 0.77 (s, 3H).

### 4.4 9α,10β-Pregest-5,7-diene-3,20-diethylene glycol ketone (1)

To a solution of compound 2 (15.18 g, 31.53 mmol) in toluene (100.00 g) was added 2,4,6-trimethylpyridine (9.20 g, 75.92 mmol) at room temperature, the reaction mixture was allowed to heat to 120 °C and stir for 15 h. The reaction solution was cooled down to room temperature and concentrated under reduced pressure. The concentrate was washed with methanol at 15 °C for 2 h. The crude products 1 were filtered, and then recrystallized in a mixture of ethyl acetate (25.00 g) and water (25.00 g). The target product 1 (10.64 g, 84.2%) is obtained after filtration. ^1^H NMR (400 MHz; CDCl_3_) *δ* 5.56 (dd, *J* = 5.9, 2.6 Hz, 1H), 5.39 (dt, *J* = 5.7, 2.7 Hz, 1H), 4.01–3.86 (m, 8H), 2.64–2.55 (m, 1H), 2.28 (dd, *J* = 15.3, 2.9 Hz, 1H), 2.19–2.02 (m, 2H), 1.92–1.80 (m, 4H), 1.78–1.70 (m, 4H), 1.66–1.54 (m, 4H), 1.46 (qd, *J* = 11.3, 6.7 Hz, 1H), 1.31 (s, 3H), 0.96 (s, 3H), 0.72 (s, 3H). ^13^C NMR (101 MHz; CDCl_3_) *δ* 140.22, 139.07, 120.01, 116.63, 111.98, 108.78, 77.34, 77.02, 76.70, 65.01, 64.44, 64.31, 63.34, 57.75, 54.40, 45.97, 42.34, 40.22, 38.85, 37.41, 36.90, 31.35, 24.50, 23.05, 22.62, 20.98, 16.17, 12.96. ESI-MS, *m*/*z*[M + H]^+^ found: 401.20.

### 4.5 3-Ethoxy-20-ethylenedioxypregna-3,5-diene (3-b)

To a solution of progesterone 4 (10.00 g, 31.80 mmol) in a mixture of solvent dichloroethane (30.00 g), triethyl orthoformate (11.00 g, 74.22 mmol) and ethylene glycol (5.50 g, 391.60 mmol) was added *p*-toluenesulfonic acid monohydrate (0.20 g, 1.05 mmol) at room temperature, the reaction mixture was allowed to heat to 40 °C and stir for 0.5 h. The reaction was cooled down to 0 °C. The crude product was filtered and the filter cake was recrystallized from ethyl acetate (30.00 g). After recrystallization and drying, the target product 3-b (9.85 g, 80.2%) was obtained. ^1^H NMR (400 MHz; CDCl_3_) *δ* 5.21 (dd, *J* = 5.0, 2.3 Hz, 1H), 5.11 (d, *J* = 1.9 Hz, 1H), 4.03–3.69 (m, 6H), 2.35–2.02 (m, 4H), 1.86–1.78 (m, 2H), 1.77–1.70 (m, 2H), 1.69–1.58 (m, 4H), 1.54 (q, *J* = 4.4 Hz, 1H), 1.44 (qd, *J* = 13.2, 4.2 Hz, 1H), 1.32–1.27 (m, 6H), 1.25 (d, *J* = 6.9 Hz, 1H), 1.23–1.18 (m, 1H), 1.16–1.00 (m, 2H), 0.98 (s, 3H), 0.80 (s, 3H). ^13^C NMR (101 MHz; CDCl_3_) *δ* 154.48, 141.06, 118.03, 112.00, 99.08, 77.33, 77.01, 76.69, 65.19, 63.22, 62.15, 58.24, 56.84, 48.32, 41.93, 39.47, 35.21, 33.87, 31.76, 31.42, 25.55, 24.58, 23.76, 23.00, 20.99, 18.98, 14.67, 12.97. ESI-MS, *m*/*z*[M + H]^+^ found: 387.25.

## Data availability

Data are present within the article and the ESI.†

## Author contributions

Conceptualization: Wang ZY, Song J, Yang YQ; data curation: Wang ZY, Huang XJ; funding acquisition: Song J, Wu HC, Yang YQ; investigation: Wang ZY, Wang Y, Jing SX; methodology: Wang ZY, Wang Y, Jing SX, Huang XJ; project administration: Wu HC, Yang YQ, Zhang B; resources: Wu HC, Yang YQ; validation: Wang ZY, Huang XJ; writing – original draft: Wang ZY; writing – review & editing: Song J, Zhang B, Yang YQ.

## Conflicts of interest

There are no conflicts to declare.

## Supplementary Material

RA-015-D5RA00109A-s001
