# Supplementary material for: Process optimizations for the synthesis of an intermediate of dydrogesterone
Source: RSC Adv. 2025 Mar 10;15(10):7656–62. doi: 10.1039/d5ra00109a (PMC11891995; doi:10.1039/d5ra00109a)
Supplement: RA-015-D5RA00109A-s001 [file RA-015-D5RA00109A-s001.pdf]

Supplementary Information (SI) for RSC Advances.
This journal is © The Royal Society of Chemistry 2025
This journal is © The Royal Society of Chemistry 2024

# Optimization of conditions for the synthesis of an intermediate of dydrogesterone

Zhongyue Wang, Yuan Wang, Shaoxiong Jing, Xiangjian Huang, Hucheng Wu, Yingquan Yang, Jian Song,* and Bao Zhang*

Tianjin University, Tianjin, China
Suzhou Entai New Materials Technology Company, Suzhou, China.

## Supplementary Information

1. Dydrogesterone synthetic route ........ 1
2. Materials and instruments ........ 2
3. Recrystallisation Process ........ 3
4. HPLC Detection Method ........ 3
5. The NMR and LC-MS Spectra ........ 4
6. Screening of Ethylene Glycol Feeding Equivalent ........ 12
7. Variation of the content of product 3 and by-products 3-a and 3-b with reaction time ........ 13
8. Comparison of the pH of different bases in toluene and aqueous solutions ........ 14
10. Stability of product 1 over time under elimination reaction conditions ........ 14
11. Stability of 7-Bromo-3,20-bis(vinylenedioxy)pregnenolone (2) ........ 14
12. HPLC spectrum of the elimination reaction mixture when NaOH or $CH_3ONa$ was used as organic base ........ 16

## 1. Dydrogesterone synthetic route

Dydrogesterone (DG) can be synthesized using four different steroid substrates.

(1) The first method uses 3β-hydroxy-9α,10β-pregna-5-en-20-one (pregnenolone) as the starting material (Fig. S1);

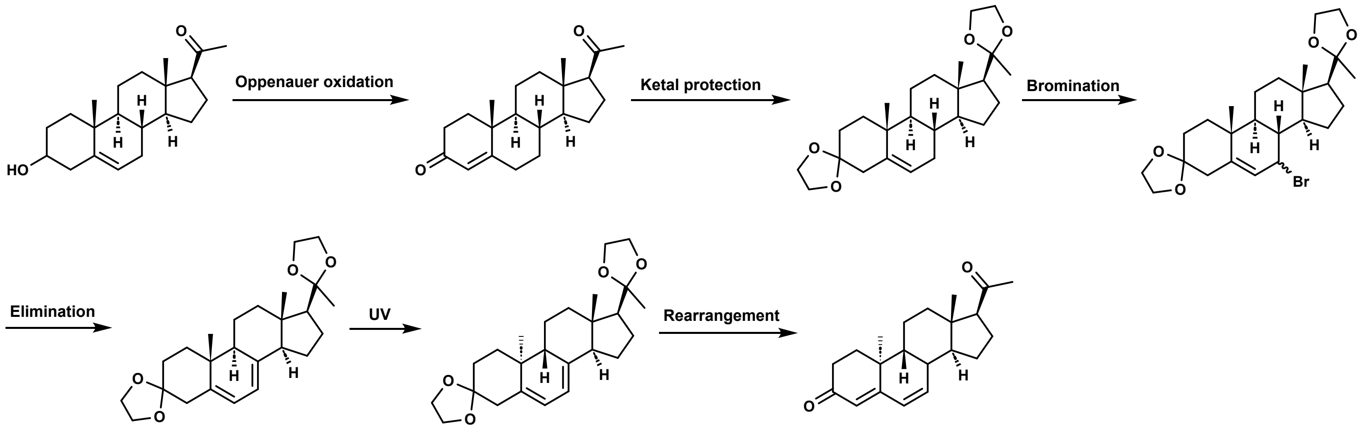


Fig. S1 Dydrogesterone synthetic route using 3β-hydroxy-9α,10β-pregna-5-en-20-one (Pregnenolone) as starting material

(2) The second method uses trans-progesterone as the starting material (Fig. S2);

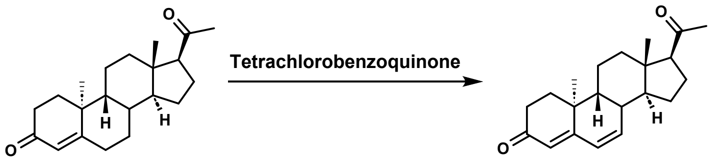


Fig. S2 Dydrogesterone synthetic route using trans-progesterone as starting material

(3) The third method uses ergosterol as the starting material (Fig. S3);

Step 1: 40 g of compound A and 500 mL of tetrahydrofuran were added to the photochemical reaction flask and the reaction was monitored by HPLC at 5-10 °C under UV high-pressure mercury lamp (500 W) irradiation for 16h.

Step 2: To a 1 L three-necked flask was added Desmartin's reagent (DMP) 105 g (0.28 mol), water 3.78 g (0.21 mol), sodium bicarbonate 35 g (0.41 mol), and 500 mL of dichloromethane (DCM) while stirring, 100 g (0.21 mol) of Compound B was added at 5 °C to 10 °C, and kept warm and stirred for half an hour.

Step 3: Add 840 mL of anhydrous ethanol in a 1 L three-necked flask, and pass dry hydrogen chloride gas (moisture should be less than 0.2%, the content of about 35%) at low temperature to prepare anhydrous ethanol/hydrogen chloride solution; add 70 g (0.145 mol) of Compound C and 700 mL of dichloromethane as well as 0.7 g of tert-butylhydroquinone (TBHQ) in a 2 L three-necked flask and react for about 1 h at controlled temperature under nitrogen protection. Add 7 g of tert-butylhydroquinone (TBHQ) under

nitrogen protection and react for about 1 h at controlled temperature. React for about 1 h under nitrogen protection. Add 7 g of tert-butylhydroquinone (TBHQ), react for 1 h at 0 °C to 10 °C, then add 840 mL of anhydrous ethanol/hydrogen chloride solution dropwise and continue to react for about 1 h. The residue of raw materials was determined to be less than 3% by TLC, and then the reaction was quenched by the addition of purified water.

Step 4: Into a 250 mL three-necked flask were added 30 g (91.3 mmol) of compound D and 150 mL of dichloromethane (DCM). Under stirring, 1.5 g (9.6 mmol) of TEMPO and 1.08 g (10.5 mmol) of sodium bromide were introduced, followed by 30 mL of a 5% aqueous sodium bicarbonate solution. The mixture was cooled to 0-5 °C under a nitrogen atmosphere, and sodium hypochlorite solution was added dropwise. The reaction was stirred for 0.5-1 h at this temperature until completion (monitored by TLC). The reaction was quenched with a sodium thiosulfate solution.

Step 5&6 (continuous operations): (1) Into a 100 mL three-necked flask were added 28 g (85.8 mmol) of compound E and 42 mL of anhydrous acetonitrile. Under stirring, 22 g (122 mmol) of cyclohexenylpiperidine (purity ≈90%) was introduced. The mixture was stirred at 40 °C under a nitrogen atmosphere until a clear solution was obtained. Acetic acid was then added, and the reaction was continued for 3-6 h. The temperature was lowered to -20 °C, and the mixture was stirred for an additional 2 h. The product was collected by filtration to afford compound F.; (2) Preparation of Copper(I) Chloride Solution. Into a 100 mL three-necked flask were added 0.42 g (4.2 mmol) of copper(I) chloride and 42 mL of DMF. The mixture was stirred at 65 °C under a nitrogen atmosphere for 1 hour and then cooled to room temperature for subsequent use. (3) Oxidation Reaction. Into a 500 mL three-necked flask were added 28 g (71.2 mmol) of compound F and 280 mL of dichloromethane (DCM). The mixture was cooled to 0-5 °C, and the pre-prepared copper(I) chloride solution was added dropwise. Dried air was bubbled through the reaction system at a flow rate of 1 L/min. The reaction was allowed to proceed for 4-8 h (monitored by TLC). Upon completion, the reaction was quenched with a 10% aqueous sulfuric acid solution. The crude product was isolated via standard work-up procedures to yield the target compound DG.

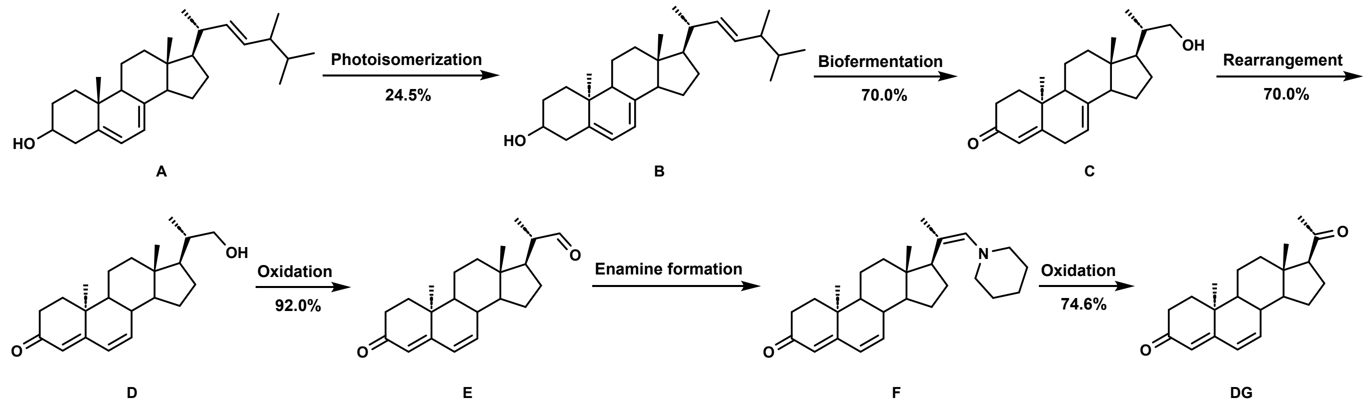


Fig. S3 Dydrogesterone synthetic route using ergosterol as starting material

(4) The fourth method uses progesterone as the starting material. It has two principal synthesis routes. One involves ketal protection, oxidation, hydrazone formation, dehydrazonation, photoisomerization, and rearrangement to prepare DG (Fig. S4);

Step 1: 5.0 L of anhydrous ethylene glycol, 3.0 g of p-toluenesulfonic acid, and 250 g (0.795 mol) of progesterone were put into a reaction flask. The reaction was heated under nitrogen protection to maintain the reaction temperature at 75-80 °C while the ethylene glycol was evaporated.

Step 2: To the reaction flask, 200 g (0.496 mol) of the product of the first step, 82 g (0.502 mol) of N-trans-phthalimide, 1.6 L of acetone and 1.6 L of ethyl acetate were added to the reaction flask, stirring was turned on, and the temperature was raised to 53±2 °C. When the raw materials were completely dissolved, 1.0 g of azodiisoheptanecarbonitrile was added, and the reaction was stirred for 6 h. The reaction was carried out with stirring.

Step 3: To the reaction flask was sequentially added 130 g of p-toluenesulfonyl hydrazide, 200 g (0.480 mol) of the reaction product of the second step, 800 ml of toluene and 1.2 L of hexane under nitrogen protection, and the reaction was warmed up and refluxed to a reaction temperature of about 75 °C. Thin-layer chromatography (TLC) was used to monitor the reaction until the end of the reaction.

Step 4: Under the protection of nitrogen, 1.0 L of anhydrous chlorobenzene and 34 g (1.47 mol) of lithium amide were added to the reaction flask, and the temperature was lowered to 15 °C-20 °C while stirring, and a solution of chlorobenzene (2 L) with 200 g (0.342 mol) of the reaction product of the third step was slowly added dropwise, keeping the temperature of the reaction solution lower than 30 °C, and then the temperature was rapidly increased to 85 °C-90 °C after the dropwise addition was completed, and the reaction was kept warm for 1 h.

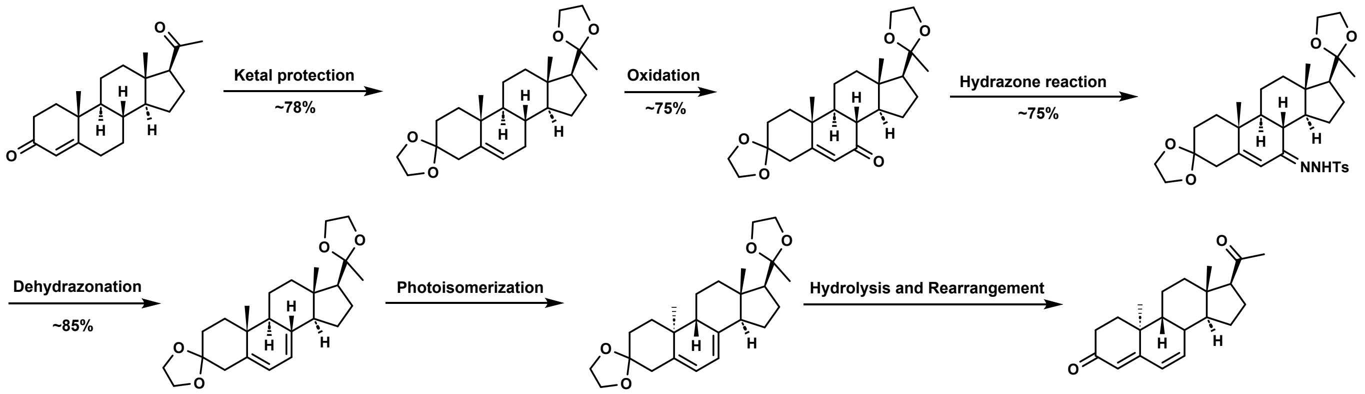


Fig. S4 Dydrogesterone synthetic route using progesterone as starting material and involving oxidation step

(5) The other route to the fourth method is through ketal protection, allyl bromination, debromination, photoisomerization, and hydrolytic rearrangement to prepare DG (Fig. S5). we choose this route to prepare DG and investigate the synthesis of intermediate **1**.

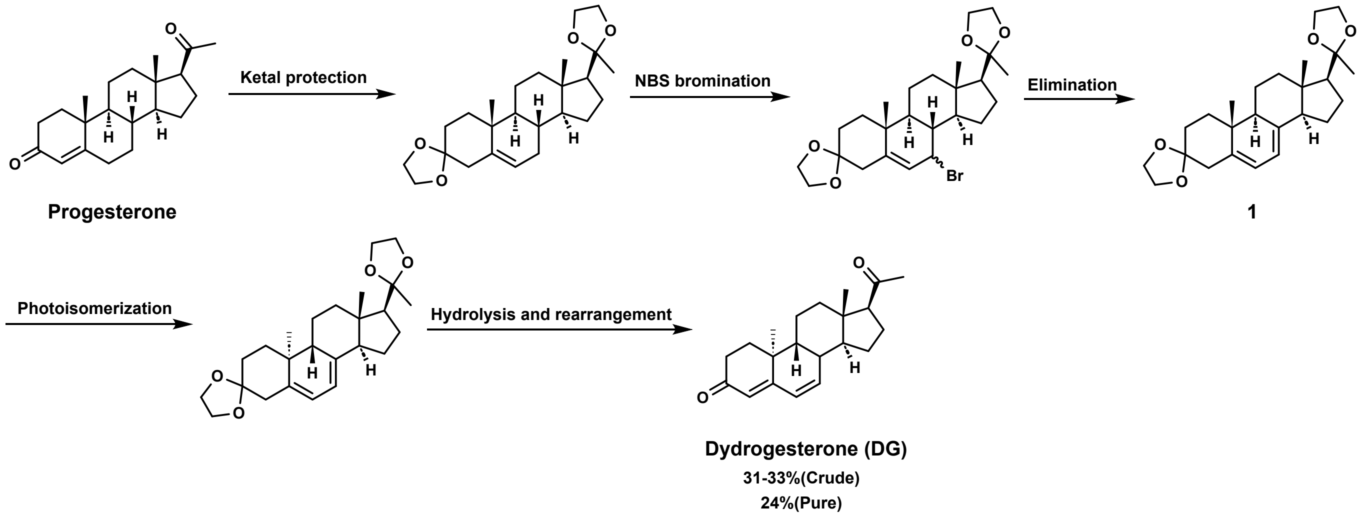


Fig. S5 Dydrogesterone synthetic route using progesterone as starting material and involving bromination step

## 2. Materials and instruments

Progesterone and 2,4,6-trimethylpyridine were purchased from Suzhou Entai New Material Technology Co. Isopropyl acetate, ethanol, cyclohexane, methyl tert-butyl ether (MTBE), toluene, methanol and methyl acetate were purchased from Shanghai Titan Technology Co. Ltd; p-toluenesulfonic acid monohydrate and azobisisobutyronitrile (AIBN) were purchased from Anhui Zesheng Technology Co. Unless otherwise noted, all commercial materials were used directly without further purification.

The reactions were monitored by a Shimadzu LC-20AT HPLC instrument. The $^{1}$H NMR and $^{13}$C NMR spectra were recorded using a Bruker Advance 400 MHz nuclear magnetic resonance (NMR) spectrometer with TMS as an internal standard. The mass spectra were recorded on a Shimadzu LC-MS 2020.

## 3. Recrystallisation Process

### 9α,10β-pregna-5-ene-3,20-diethylene glycol ketone (3)

A flask charged with the crude filter cake and ethyl acetate (60.00g) was heated up to 75-80 °C and stirred at reflux for 1 h. Then the solution was cooled down to room temperature and stirred for 2 h. The target crude product **3** was then filtered, and the filter cake was washed with water and ethyl acetate, and dried in vacuum to obtain 22.74 g of compound **3**, with a yield of 88.82%.

### 3,20-Bis(vinylenedioxy)pregnane-5,7-diene (1)

A flask charged with the crude filter cake, ethyl acetate (24.18 g) and water (24.18 g) was heated up to 75-80 °C and stirred at reflux for 2 h. Then the solution was cooled down to room temperature and stirred for 2 h. The target crude product **1** was then filtered, and the filter cake was washed with water and ethyl acetate, and dried in vacuum to obtain 10.64 g of compound **1**, with a yield of 84.23%.

## 4. HPLC Detection Method

### 9α,10β-pregna-5-ene-3,20-diethylene glycol ketone (3)

The separation was carried out on an octadecylsilane-bonded silica gel as filler ($C_{18}$ chromatographic column, 4.6 mm×150 mm×3 μm), with the mobile phase of water (A): acetonitrile (B), gradient elution (Table S1) at a flow rate of 1.0 mL/min, the detection wavelength of 210 nm, and the column temperature of 40 °C, with an injection volume of 10 μL.

Table S1 HPLC detection gradient of 9α,10β-pregna-5-ene-3,20-diethylene glycol ketone (**3**) reaction solution and products

| **Time/min** | **Mobile phase A/%** | **Mobile phase B/%** |
|---|---|---|
| 0 | 40 | 60 |
| 40 | 5 | 95 |
| 50 | 5 | 95 |
| 50.1 | 40 | 60 |
| 60 | 40 | 60 |

### 7-Bromo-3,20-bis(vinylenedioxy)pregnenolone (2)

The separation was carried out on an octadecylsilane-bonded silica gel as filler ($C_{18}$ column, 4.6 mm×150 mm×5 μm), with the mobile phases of buffer solution (A) and acetonitrile (B), the buffer solution was 0.005 mol/L aqueous potassium dihydrogen phosphate, gradient elution (Table S2), flow rate was 1.5 mL/min, the detection wavelength was 210 nm, the column temperature was 40 °C, and the injection volume was 10 μL. The column temperature was 40 °C, and the injection volume was 10 μL.

Table S2 HPLC detection gradient of 7-Bromo-3,20-bis(vinylenedioxy)pregnenolone (2) reaction solution and products

| Time/min | Mobile phase A/% | Mobile phase B/% |
|---|---|---|
| 0 | 70 | 30 |
| 30 | 25 | 75 |
| 40 | 25 | 75 |
| 40.1 | 70 | 30 |
| 50 | 70 | 30 |

### 3,20-Bis(vinylenedioxy)pregnane-5,7-diene (1)

The separation was carried out on an octadecylsilane-bonded silica gel as filler ($C_{18}$ chromatographic column, 4.6 mm×150 mm×5 μm), with the mobile phase of water (A):acetonitrile (B), gradient elution (Table S3) at a flow rate of 1.0 mL/min, the detection wavelengths of 210 nm and 254 nm, the column temperature of 40 °C, and the injection volume of 10 μL.

Table S3 HPLC detection gradient of 3,20-Bis(vinylenedioxy)pregnane-5,7-diene (1) reaction solution and products

| Time/min | Mobile phase A/% | Mobile phase B/% |
|---|---|---|
| 0 | 40 | 60 |
| 40 | 5 | 95 |
| 50 | 5 | 95 |
| 50.1 | 40 | 60 |
| 60 | 40 | 60 |

## 5. The NMR and LC-MS Spectra

Progesterone: $^{1}$H NMR (400 MHz, Chloroform-d) δ 5.83 – 5.61 (m, 1H), 2.53 (t, *J* = 9.0 Hz, 1H), 2.47 – 2.23 (m, 4H), 2.23 – 2.14 (m, 1H), 2.12 (d, *J* = 1.0 Hz, 3H), 2.04 (ddd, *J* = 18.4, 8.2, 3.6 Hz, 2H), 1.85 (dp, *J* = 12.3, 2.9 Hz, 1H), 1.76 – 1.38 (m, 8H), 1.27 (ddd, *J* = 17.2, 11.7, 5.6 Hz, 1H), 1.18 (s, 3H), 1.01 (ddt, *J* = 27.0, 15.6, 6.3 Hz, 2H), 0.66 (s, 3H).

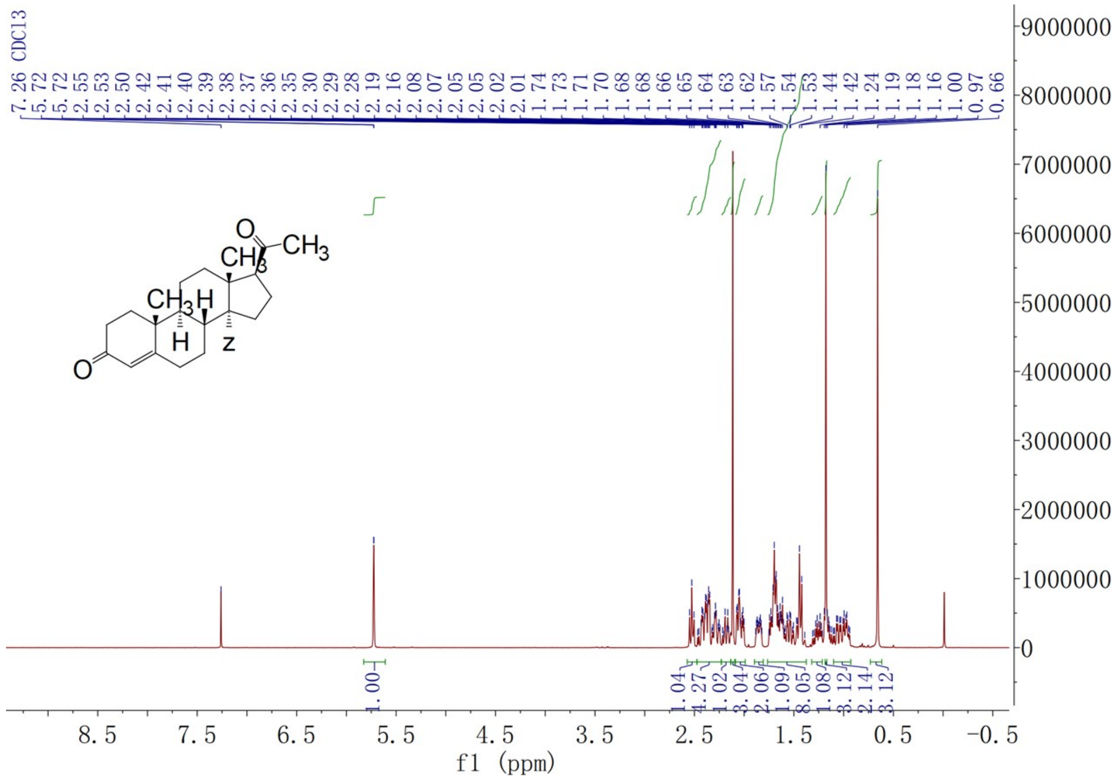

Fig. S6 ¹H NMR spectrum of Progesterone

Progesterone: $^{13}C$ NMR (101 MHz, $CDCl_3$) δ 140.18, 122.09, 111.99, 109.48, 77.37, 77.05, 76.73, 65.16, 64.44, 64.21, 63.23, 58.17, 56.59, 49.68, 41.80, 39.42, 36.66, 36.32, 31.66, 31.45, 31.07, 24.56, 23.84, 22.99, 20.84, 18.88, 12.87.

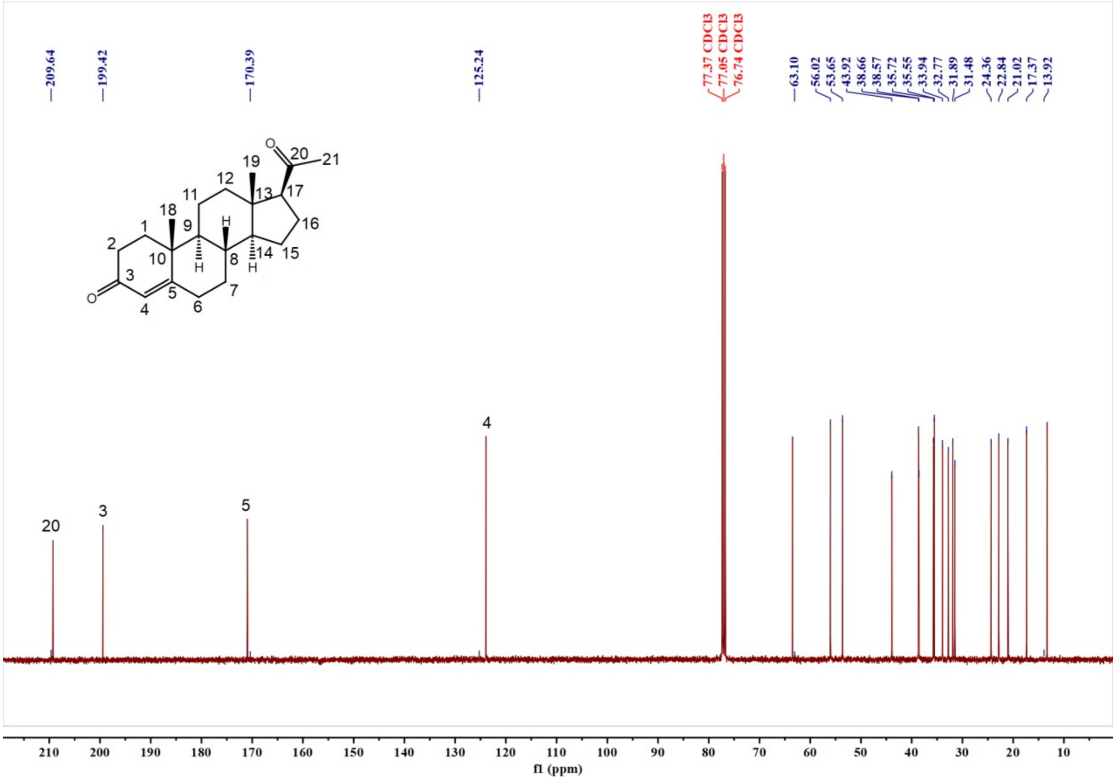


Fig. S7 $^{13}$C NMR spectrum of Progesterone

Progesterone: ESI-MS, m/z[M+H]$^+$ found: 315.20.

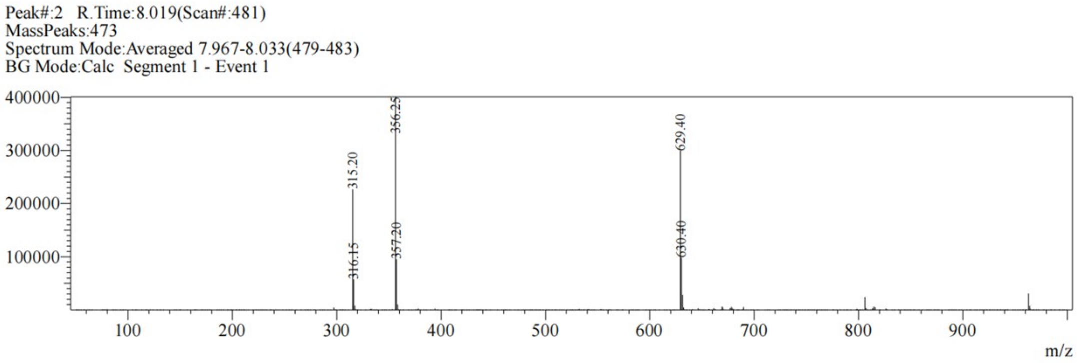


Fig. S8 LC-MS plot of Progesterone

Compound **3**: $^{1}$H NMR and $^{13}$C NMR spectrum.

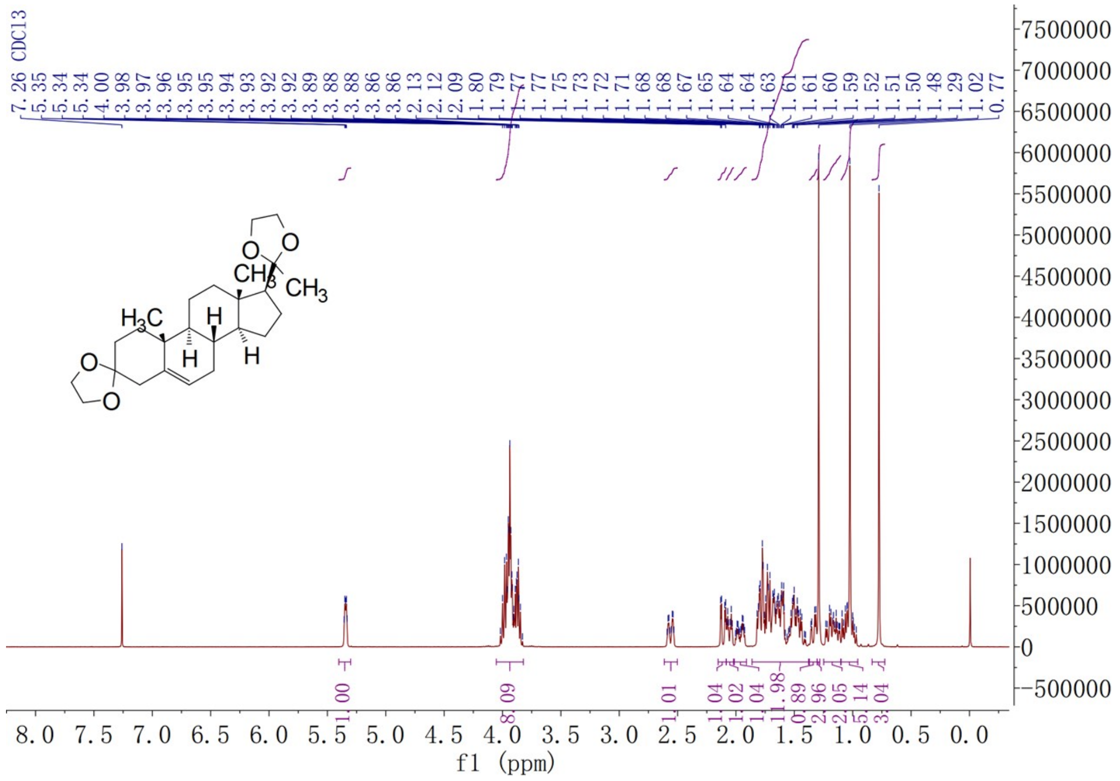

Fig. S9 $^{1}$H NMR spectrum of Compound **3**

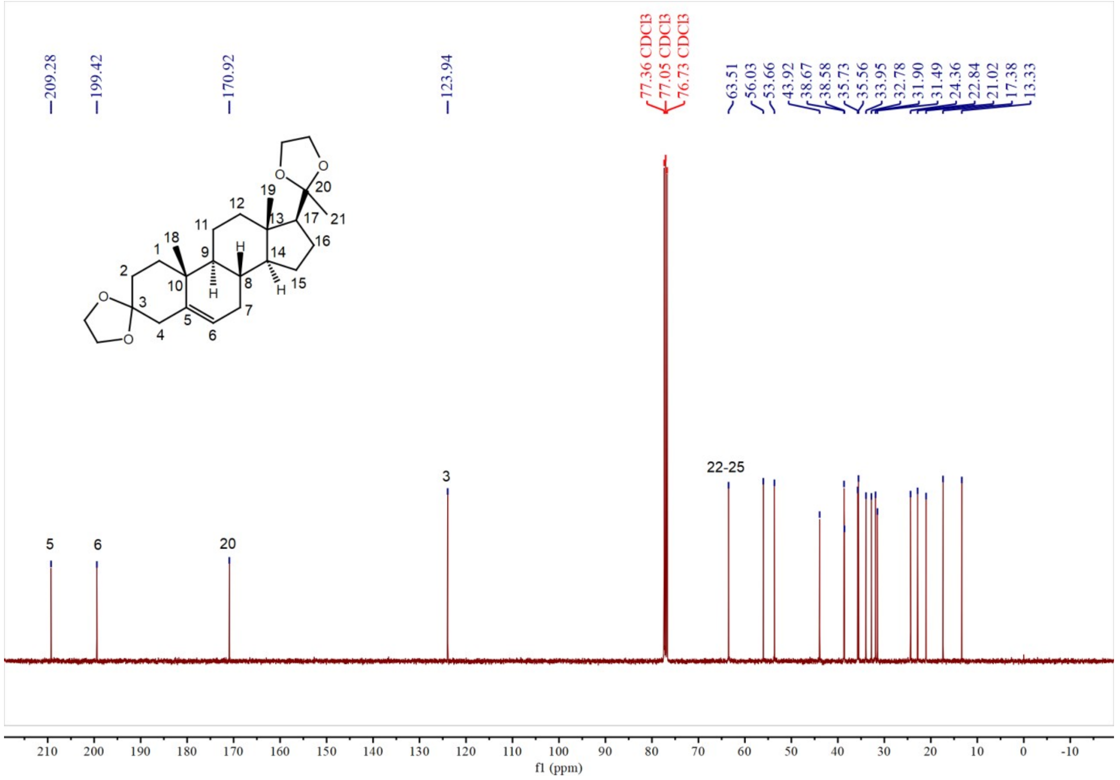

Fig. S10 $^{13}$C NMR spectrum of Compound **3**

Compound **3**: ESI-MS, m/z $[M+H]^+$ found: 403.30.

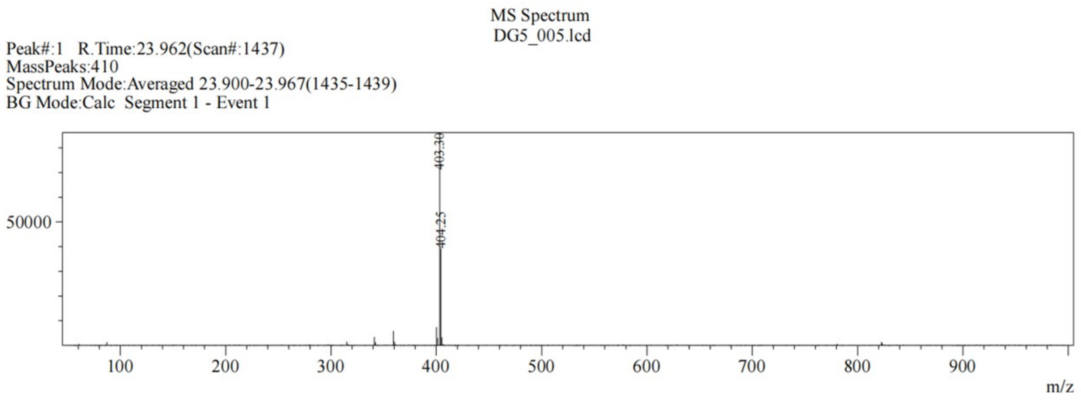


Fig. S11 LC-MS plot of Compound **3**

Compound **2**: $^{1}$H NMR spectrum.

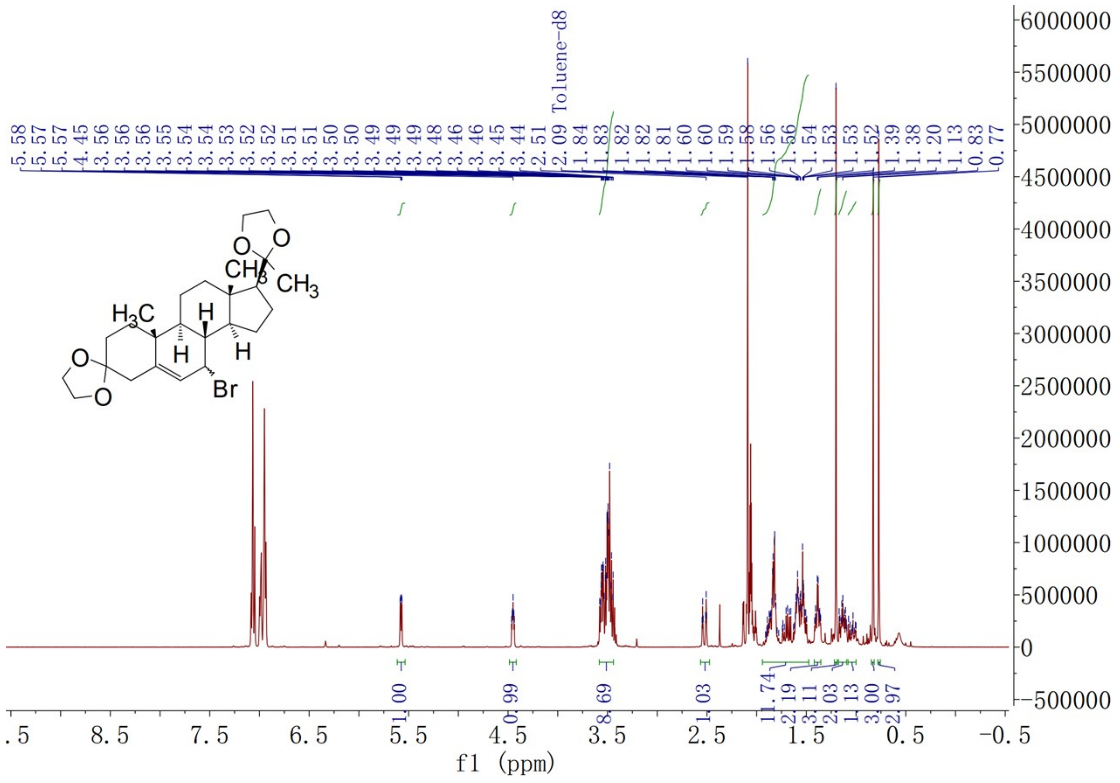


Fig. S12 $^{1}$H NMR spectrum of Compound **2**

Compound **2**: ESI-MS, m/z $[M+H]^+$ found: 481.20. Compound 2 was difficult to stabilize at room temperature and was debrominated during LC-MS detection, with a relative molecular mass of 401.20, consistent with compound **1.** The calculated relative molecular mass of compound **2** was 482.11, which in combination with the $^{1}$H NMR spectrum was confirmed as compound **2**.

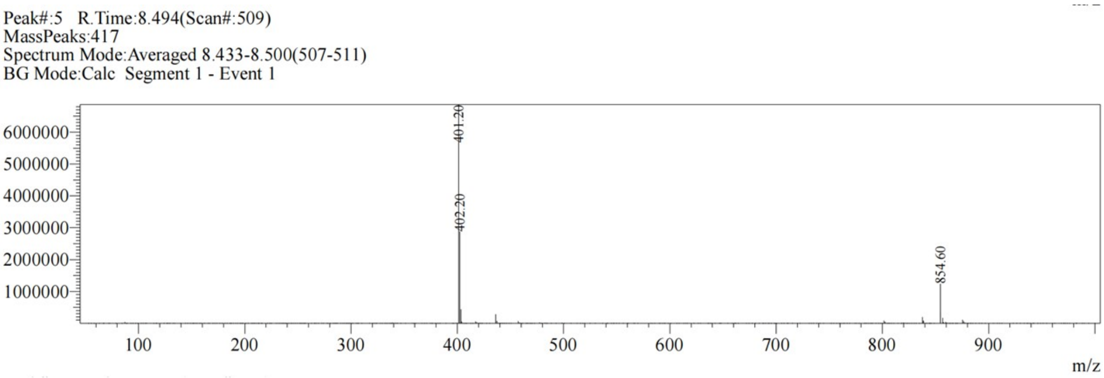


Fig. S13 LC-MS plot of Compound **2**

Compound **1:** $^{1}$H NMR and $^{13}$C NMR spectrum.

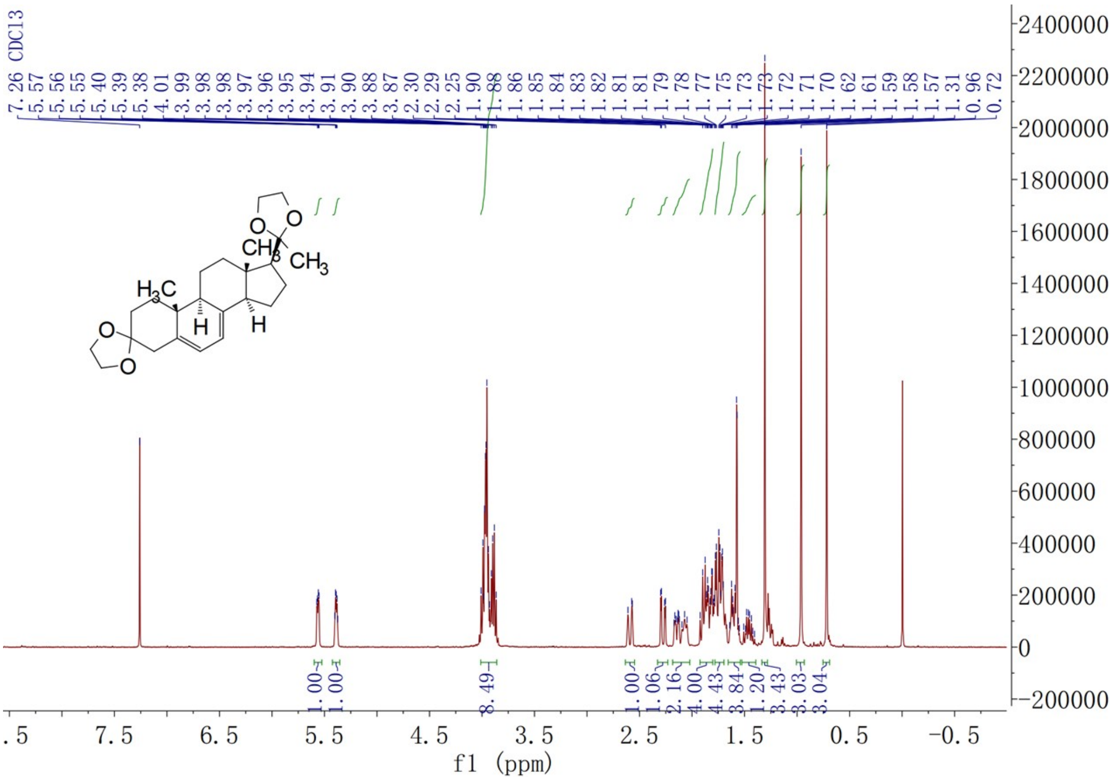


Fig. S14 $^{1}$H NMR spectrum of Compound **1**

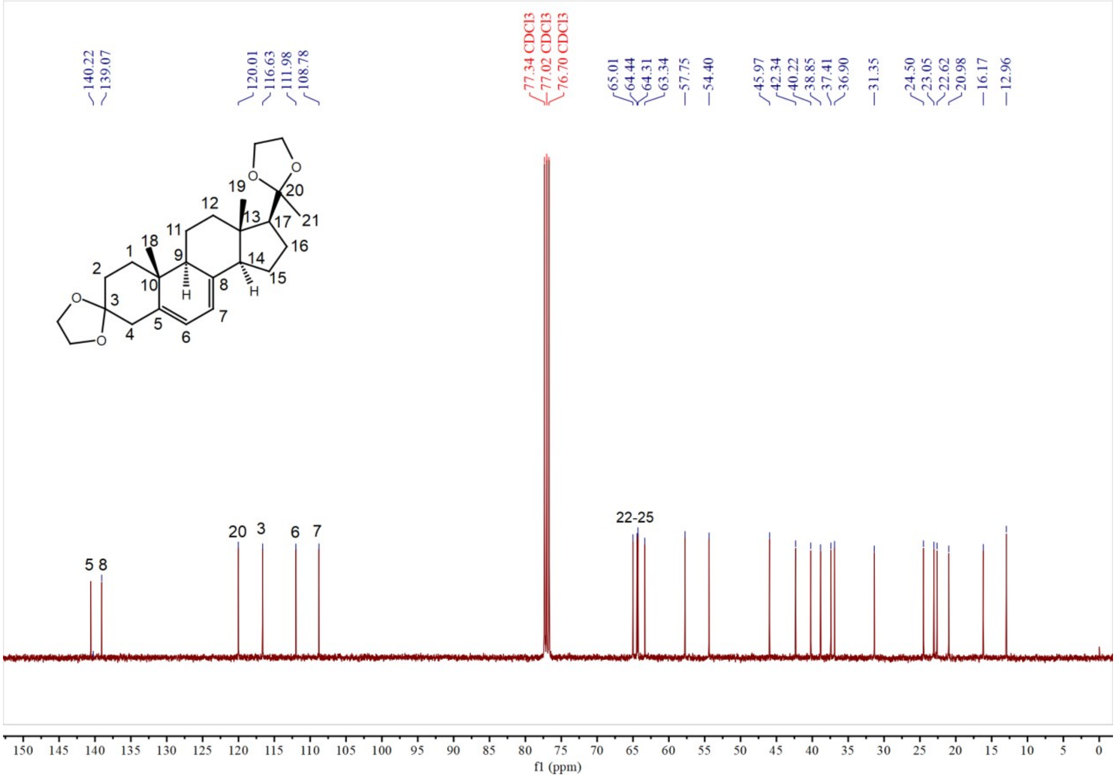

Fig. S15 $^{13}C$ NMR spectrum of Compound **1**

Compound **1**: ESI-MS, m/z[M+H]$^+$ found: 401.20.

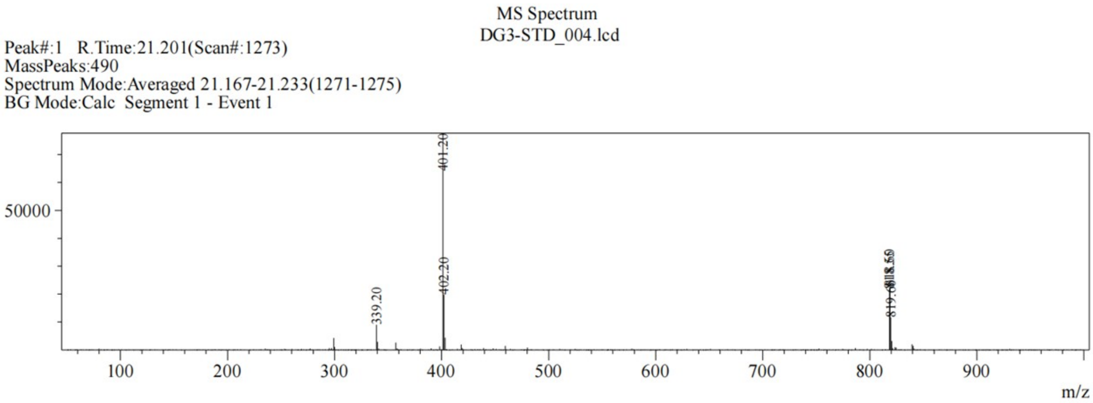


Fig. S16 LC-MS plot of Compound **1**

Compound **3-b**: $^1H$ NMR and $^{13}C$ NMR spectrum.

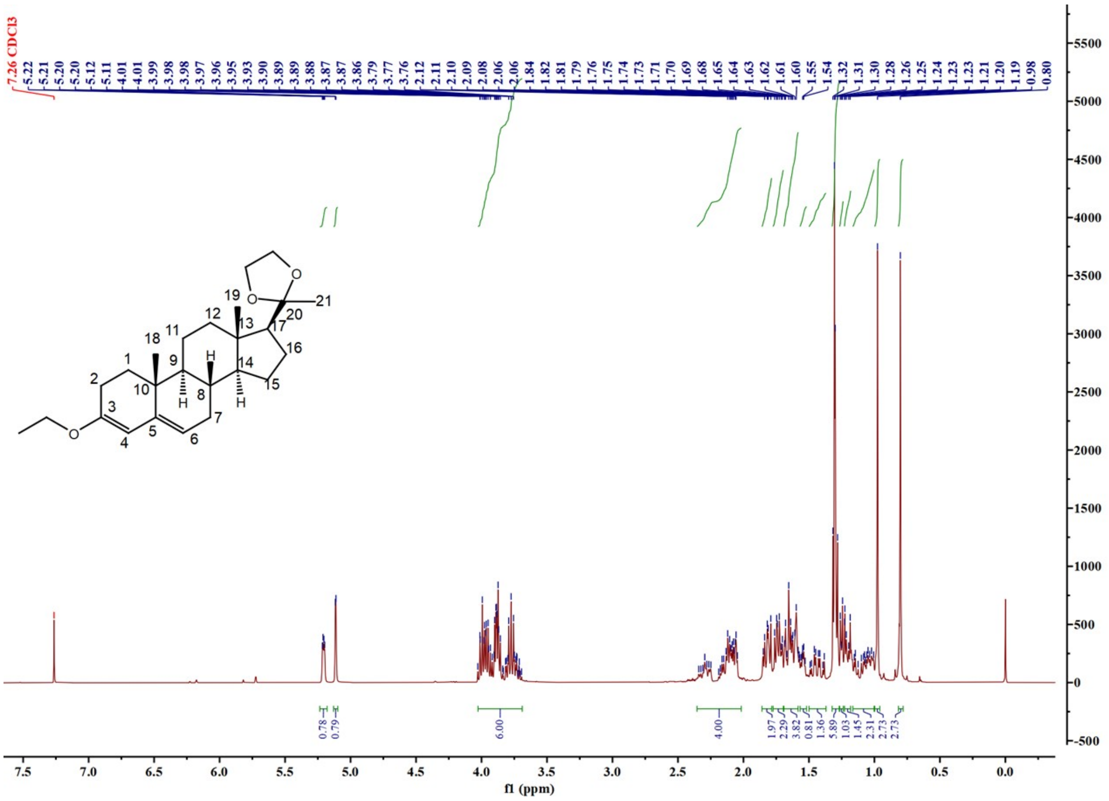


Fig. S17 $^{1}$H NMR spectrum of Compound **3-b**

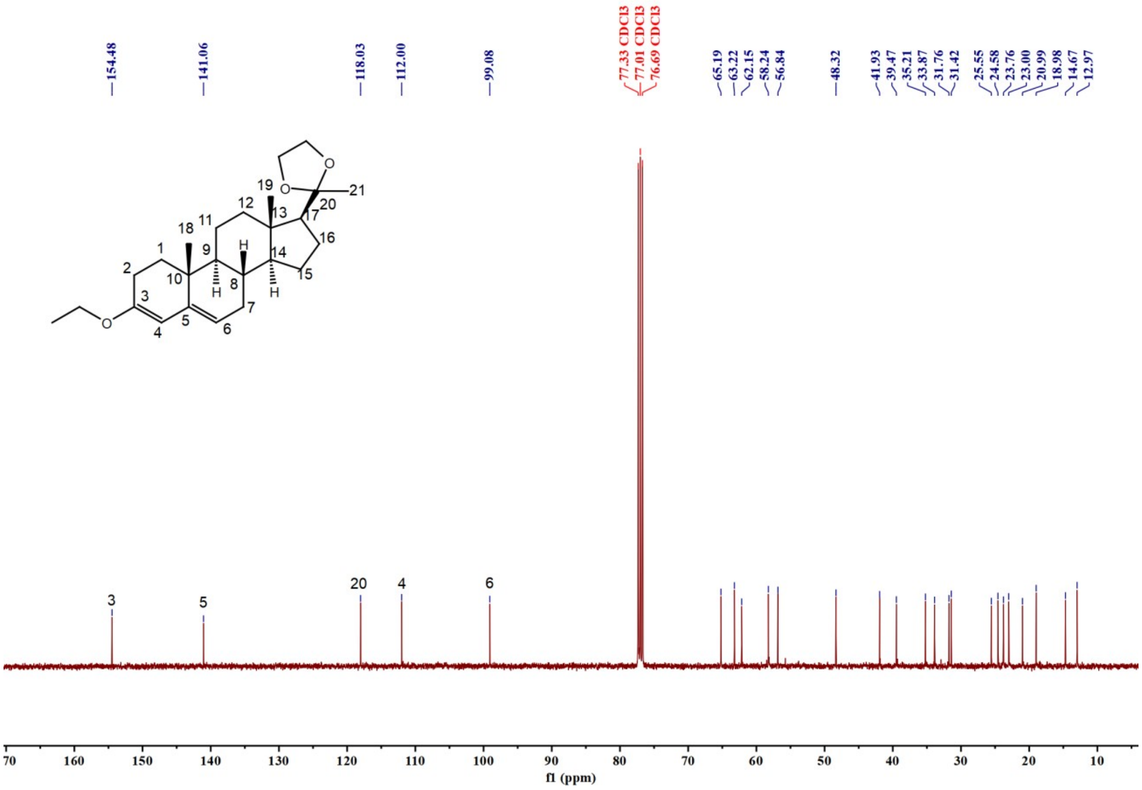


Fig. S18 $^{13}$C NMR spectrum of Compound **3-b**

Compound **3-b**: ESI-MS, m/z$[M+H]^+$ found: 387.25.

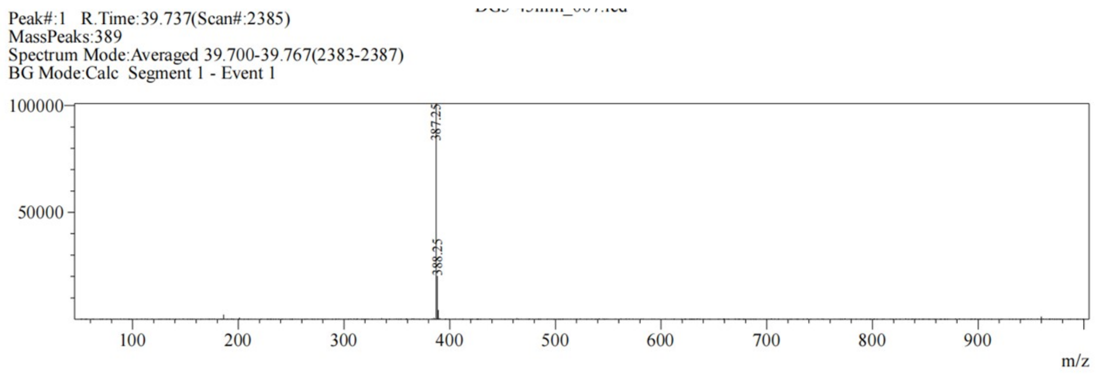


Fig. S19 LC-MS plot of Compound **3-b**

## 6. Screening of Ethylene Glycol Feeding Equivalent

Table S4 demonstrated a comparison of the yields of **3** and **3-b** under different molar ratio conditions. As stated in the manuscript, when the mole ratio of n(EG):n(4) was less than 3:1, the ketal protection reaction was insufficient, resulting in the formation of the main by-product 3-b (Table S4, Entries 1-2). The HPLC spectra of the reaction solution at a molar ratio of n(EG):n(4) of 2:1 further confirmed the above conclusion (Fig. S20).

Table S4 Screening of Ethylene Glycol Feeding Equivalent

| Entry[a] | EG:TEOF:4 (equiv.) | Temp. | Time | Yield of 3[b] | Yield of 3-b[b] |
|---|---|---|---|---|---|
| 1 | 2.0:2.3:1 | 40 °C | 15 h | 35.1% | 44.4% |
| 2 | 3.0:2.3:1 | 40 °C | 15 h | 69.4% | 6.3% |
| 3 | 4.0:2.3:1 | 40 °C | 15 h | 71.4% | 3.6% |
| 4 | 5.0:2.3:1 | 40 °C | 15 h | 76.7% | 3.1% |
| 5 | 6.0:2.3:1 | 40 °C | 15 h | 83.8% | 3.2% |
| 6 | 7.0:2.3:1 | 40 °C | 15 h | 82.9% | 2.6% |
| 7 | 8.0:2.3:1 | 40 °C | 15 h | 81.3% | 2.1% |
| 8 | 9.0:2.3:1 | 40 °C | 15 h | 78.6% | 1.1% |

[a]Conditions: compound 4 (63.60 mmol, 1 equiv.), TEOF (148.25 mmol, 2.3 equiv.), TsOH·$H_2O$ (2.10 mmol, 0.03 equiv.). [b]Isolated yield after workup.

<Chromatogram>

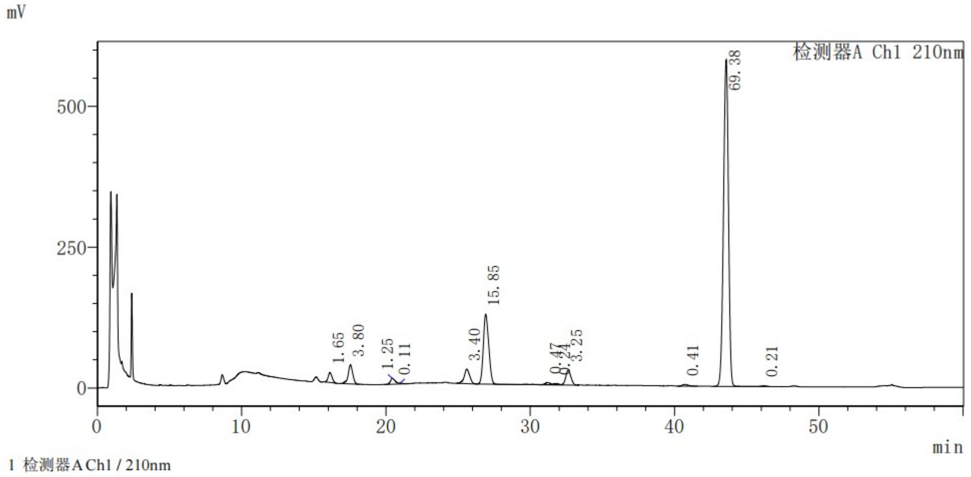


1 检测器A Ch1 / 210nm

<Peak Table>

检测器A Ch1 210nm

| Peak# | Name | Ret. Time | Height | Area | Area% |
|---|---|---|---|---|---|
| 1 | | 16.115 | 17619 | 329189 | 1.646 |
| 2 | | 17.532 | 33889 | 758882 | 3.796 |
| 3 | | 20.457 | 10366 | 249250 | 1.247 |
| 4 | | 20.978 | 1580 | 22685 | 0.113 |
| 5 | 3-a | 25.600 | 25847 | 678859 | 3.395 |
| 6 | 3 | 26.911 | 124132 | 3169024 | 15.850 |
| 7 | | 31.198 | 3920 | 93151 | 0.466 |
| 8 | | 31.817 | 2147 | 48065 | 0.240 |
| 9 | | 32.622 | 26336 | 649415 | 3.248 |
| 10 | | 40.709 | 3009 | 82784 | 0.414 |
| 11 | 3-b | 43.576 | 579869 | 13871625 | 69.379 |
| 12 | | 46.151 | 1467 | 41127 | 0.206 |
| 总计 | | | 830182 | 19994057 | 100.000 |

Fig. S20 HPLC spectrum of the reaction solution ( n(EG):n(4)=2:1 )

## 7. Variation of the content of product 3 and by-products 3-a and 3-b with reaction time

Table S5 Variation of the content of product **3** and by-products **3-a** and **3-b** with time

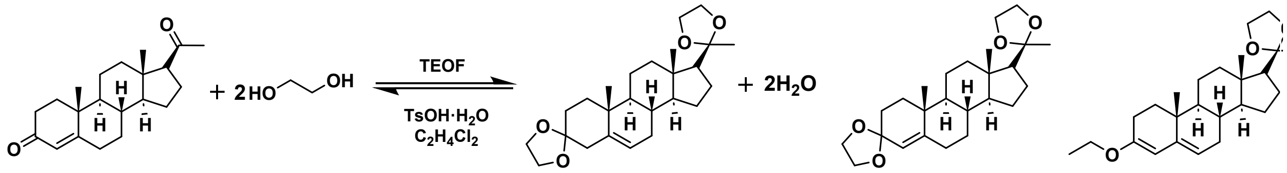


**4** **EG** **3** **3-a By-product** **3-b By-product**

| Entry[a] | Time | 3, wt% | 3-a, wt% | 3-b, wt% |
|---|---|---|---|---|
| 1 | 3 h | 73.87 | 7.61 | 8.57 |
| 2 | 6 h | 77.70 | 5.68 | 5.72 |
| 3 | 9 h | 81.36 | 4.81 | 5.40 |
| 4 | 12 h | 83.38 | 3.92 | 5.07 |
| 5 | 15 h | 82.90 | 2.06 | 1.95 |
| 6 | 18 h | 81.76 | 4.27 | 4.45 |
| 7 | 21 h | 82.92 | 4.46 | 4.77 |

[a]Conditions: compound **4** (63.60 mmol, 1 equiv.), EG (381.02 mmol, 6 equiv.), TEOF (148.25 mmol, 2.3 equiv.), TSOH·$H_2O$ (2.10 mmol, 0.03 equiv.).

## 8. Comparison of the pH of different bases in toluene and aqueous solutions

Table S6 Comparison of the pH of different bases in toluene and aqueous solutions

| fluid \ base | **DBU** | **TEA** | **DIPEA** | **2,6-dimethylpyridine** | **2,4,6-trimethylpyridine** |
|---|---|---|---|---|---|
| toluene | 13 | 13-14 | 13 | 8-9 | 8-9 |
| water | 13.50 | 12.03 | 11.65 | 9.70 | 7.39 |

## 10. Stability of product 1 over time under elimination reaction conditions

Table S7 Stability of **1** over time under elimination reaction conditions

| **Time/h** | **1:1-a (HPLC area%)** |
|---|---|
| 0 | 6.47 |
| 1 | 6.24 |
| 2 | 6.11 |
| 3 | 5.97 |

## 11. Stability of 7-Bromo-3,20-bis(vinylenedioxy)pregnenolone (2)

Compound **2** has poor stability and is easily hydrolyzed when exposed to air at room temperature or above. Also the bromine substituent of **2** is unstable and easily dissociated. The $^1$H NMR of the decomposition product is shown in Fig. S21. The compound lacks the characteristic multiplet peaks at 3-4 ppm ascribed to the ethylene glycol protecting group and has singlets around 5.7 and 6.2 ppm, suggesting the presence of a 5,7-conjugated diene structure. Therefore, it is presumed that the structural formula of the impurity after the decomposition is shown in Fig. S22.

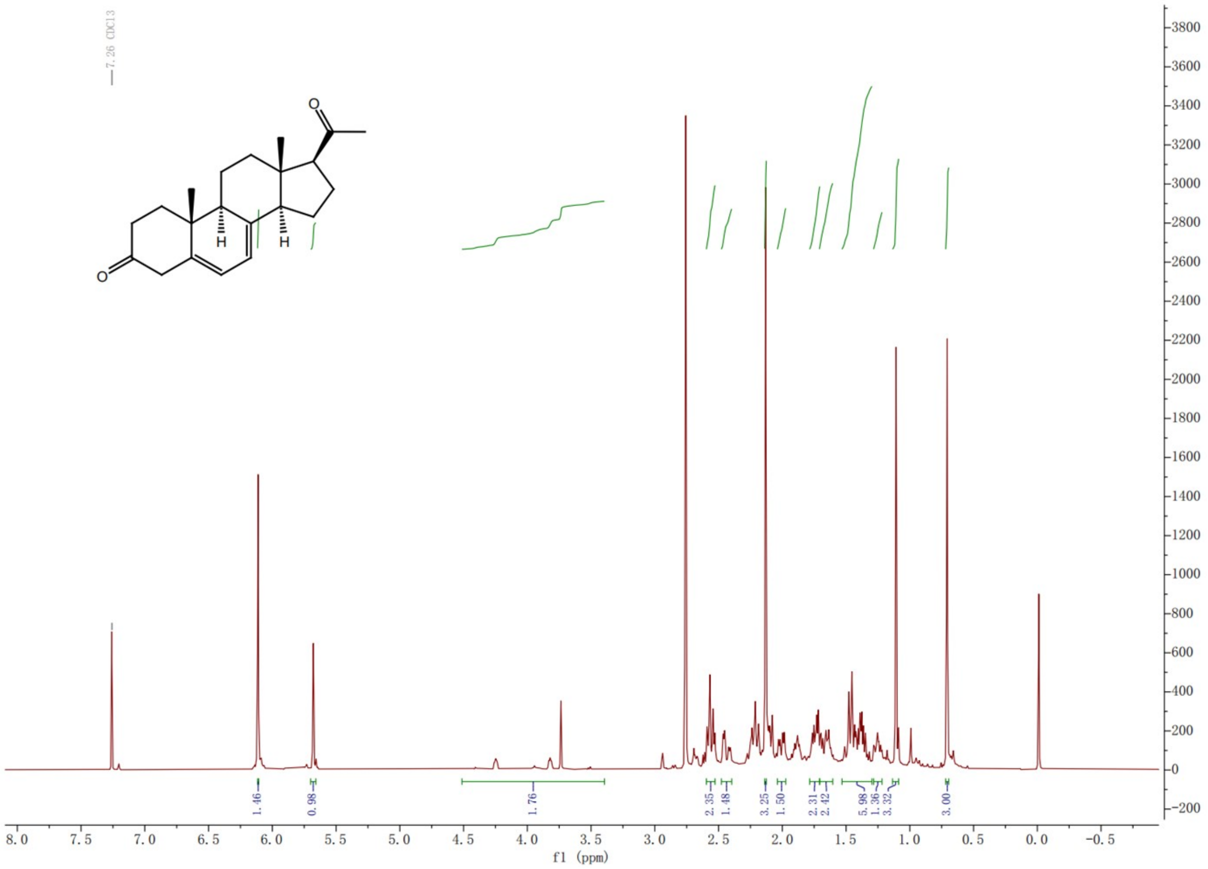


Fig. S21 $^{1}$H NMR spectrum of impurity **2-a**

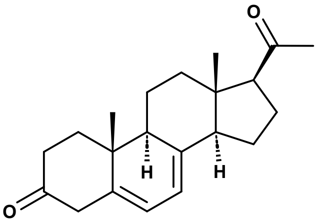


Fig. S22 Molecular structure of impurity **2-a**

Because of the instability of bromide **2** and its analogs, the analysis and separation of by-products from the bromination reaction are challenging. Structural decomposition or isomerization may occur during the LC-MS detection process. The highest content of by-products in the bromination reaction is suspected to be the dibromide **2-b**, and we have speculated on its structure through LC-MS (Fig. S23 and S24).

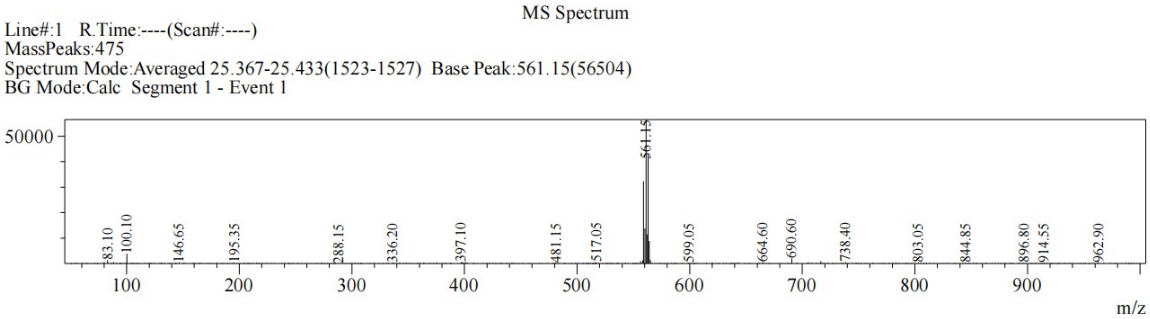


Fig S23 LC-MS spectrum of the dibromo substitute **2-b**

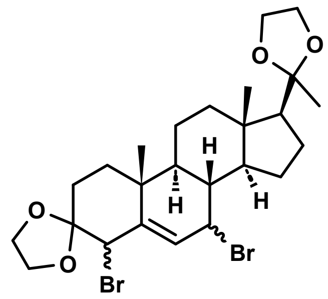


Fig. S24 Putative structure of the dibromo substituent **2-b**

## 12. HPLC spectrum of the elimination reaction mixture when NaOH or $CH_3ONa$ was used as organic base

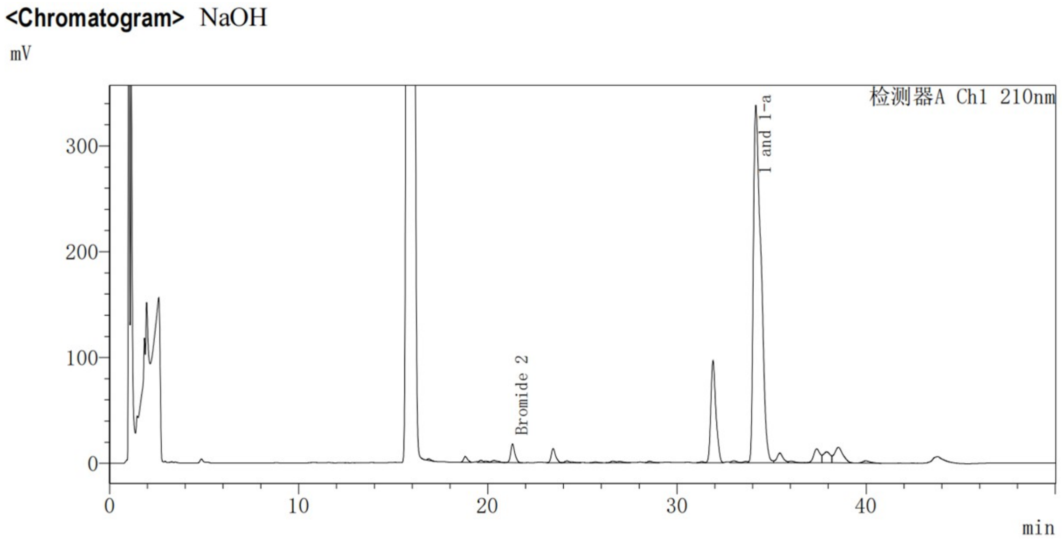


Fig. S25 HPLC spectrum of the elimination reaction solution in the presence of NaOH

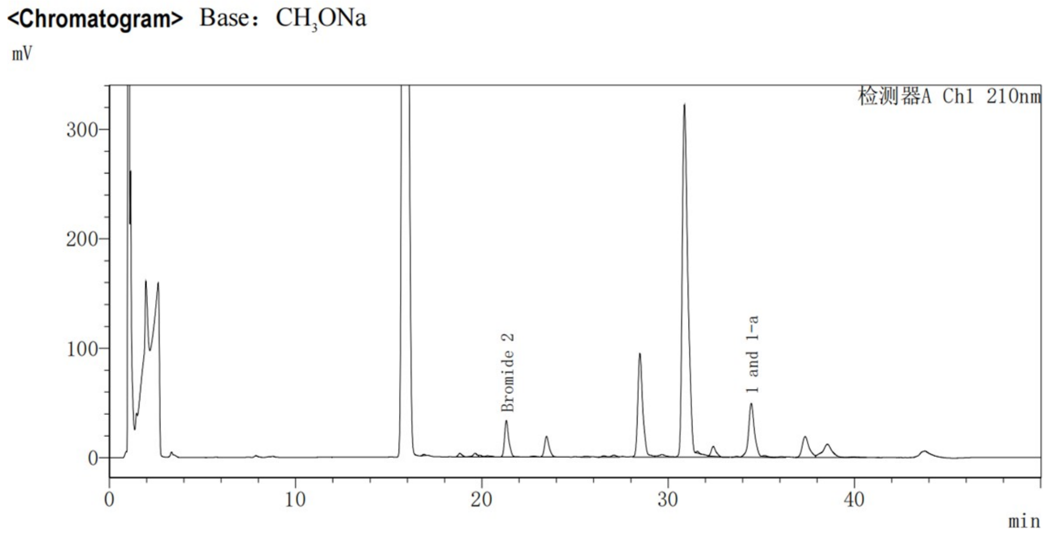


Fig. S26 HPLC spectrum of the elimination reaction solution in the presence of NaOH
